# Pregnancy options counseling in medical education and professionalism development

**DOI:** 10.1016/j.xagr.2026.100656

**Published:** 2026-05-19

**Authors:** Lucy D Brown, Carli A King, Leah J Peipert, Jeffrey F Peipert, Julianne Stout, Amy Caldwell

**Affiliations:** 1 Department of Obstetrics and Gynecology, Indiana University School of Medicine, Indianapolis, IN, USA (Brown, King, LJ Peipert, JF Peipert, Stout, and Caldwell); 2 Department of Gynecology and Obstetrics, Johns Hopkins School of Medicine, Baltimore, MD, USA (Brown and JF Peipert); 3 University of Utah School of Medicine, Salt Lake City, UT, USA (King and JF Peipert); 4 Brown University School of Medicine, Providence, RI, USA (LJ Peipert and JF Peipert); 5 Department of Obstetrics and Gynecology, University of Vermont School of Medicine, Burlington, VT, USA (JF Peipert)

**Keywords:** abortion, abortion referral, medical education, pregnancy options counseling, professionalism, referral

## Abstract

**BACKGROUND:**

Recent changes in both federal and state-level legislation have made accessing abortion care challenging, if not impossible, for some patients. As a result, patients’ reliance on comprehensive, accurate pregnancy options counseling from trusted health providers has become critical to patient access and health equity. The current generation of medical students will play an increasingly important role in counseling patients on pregnancy options.

**OBJECTIVE:**

To analyze the utility of a novel pregnancy options counseling curriculum by examining preclinical medical student perspectives on professional identity development as elicited by the curriculum.

**STUDY DESIGN:**

This qualitative study investigated second-year medical students’ perspectives on pregnancy options counseling after attending a virtual “Pregnancy Options Counseling Panel.” The panel consisted of an interactive, case-based discussion of a patient diagnosed with an unintended pregnancy. After the panel, students submitted a prompt-driven reflection on professional identity development as it applies to pregnancy options counseling. The responses were coded using the AAMC Physician Competencies for Professionalism to identify themes and insights; competencies included “Physician Accountability,” “Compassion, Integrity, Respect,” “Patient Needs,” “Patient Autonomy,” “Sensitivity to Diverse Populations,” and “Commitment to Ethical Principles.” Coding was performed using the software Dedoose.

**RESULTS:**

348 student responses were included in this analysis. The most common codes applied were “Physician Accountability” (n=416), “Compassion, Integrity, Respect” (n=201), and “Patient Needs” (n=178). Inter-reviewer reliability tests showed significant agreement for all codes (k>0.63). The majority of student responses demonstrated accountability to patients while emphasizing the importance of compassionate, nonjudgmental care.

**CONCLUSION:**

Education on pregnancy options counseling is useful in professional identity development for medical students. The curriculum challenged students to reconcile their personal beliefs with their professional obligations, and most student responses were thoughtful and empathic. Pregnancy options counseling serves as an ideal vehicle for introducing value conflict as a measure of professionalism, while simultaneously improving students’ medical knowledge on pregnancy termination and family planning.

**AJOG Global Reports at a Glance**

**Why was this study conducted?**

To assess the utility of pregnancy options counseling as a tool for professional identity formation.

**Key findings**

Student responses were consistent with the AAMC Physician Competencies for Professionalism, especially “Physician Accountability”, “Compassion, Integrity, Respect”, and “Patient Needs”.

**What does this add to what is known?**

Reinforces medical students’ desire for comprehensive reproductive health education. Provides a framework for future curriculum standardization. Demonstrates the utility of pregnancy options counseling as a tool to facilitate professional identity development.

## Introduction

Pregnancy options counseling is an important skill for physicians, as unintended and unplanned pregnancies are common and frequently detected in various clinical contexts. Many patients will ultimately decide against the continuation of pregnancy and/or parenting. Approximately 1 in 4 women will seek an abortion in their lifetime,^1^ and about 18,000 children are relinquished for adoption annually.^2^ Education on pregnancy options counseling is increasingly relevant, as several states have restricted abortion care access. The number of unsafe, self-managed abortions is expected to rise in response to restricted abortion care access; thus, patients’ reliance on comprehensive, accurate pregnancy options counseling from trusted figures will become even more important. Americans consistently rate the honesty and ethical standards of medical doctors above those of police officers, judges, lawyers, and members of Congress, according to a Gallup survey by Brenan et al.^3^ Thus, physicians play a major role in providing reliable, trustworthy counseling.

As reproductive care becomes increasingly politicized and restricted, the next generation of physicians must be equipped to counsel patients on pregnancy options.^4^ Regardless of intended specialty, every physician will encounter pregnant patients, and even those with conscientious objection to aspects of reproductive healthcare should be able to respond in a clinical setting where their patient unexpectedly finds out that they are pregnant.

**Figure 1:**
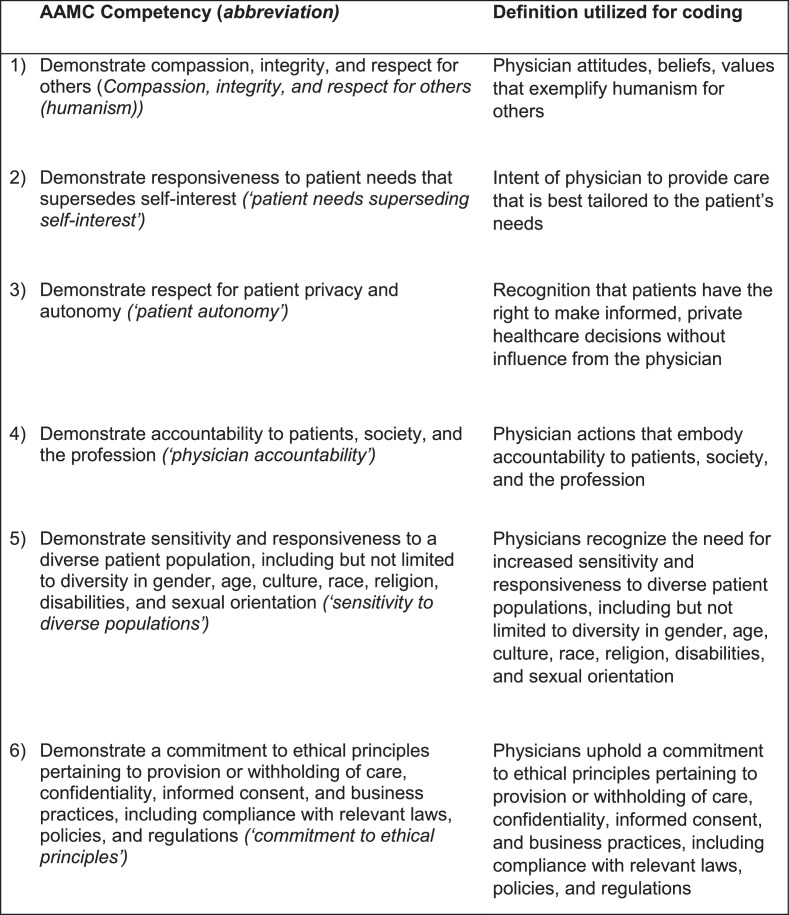
AAMC professionalism competencies^6^ followed by each respective definition that the researchers used for consistent coding

The ability to reconcile personal beliefs with professional duties is a skill that traverses all medical specialties. Pregnancy options counseling serves as an ideal vehicle to aid the development of professionalism in the preclinical space, as it introduces value conflicts between personal beliefs and obligations of professionalism.^5^ The American Association of Medical Colleges (AAMC) has identified 6 core physician competencies for professionalism that all medical students should demonstrate before entering residency: “Compassion, integrity and respect,” “Accountability,” “Autonomy,” “Patient needs,” “Sensitivity,” and “Commitment to ethical principles”^6^ (Figure 1). Prior qualitative studies have found that exploring attitudes surrounding abortion is an effective method to determine professionalism traits as outlined by the AAMC.^1^^,^^5^

Brown. Pregnancy options counseling in medical education. J Obstet Gynecol 2026.

This study evaluates a novel preclinical curriculum on pregnancy options counseling that consisted of asynchronous didactic modules and an interactive panel with healthcare professionals covering abortion, adoption, and pregnancy continuation. We hypothesized that the pregnancy options counseling curriculum would promote student reflection and engagement with the AAMC core physician competencies for professionalism. This cross-sectional view of medical students’ perspectives on pregnancy options counseling early in medical training may guide future education and identify educational gaps in reproductive counseling.

### Objective

We aim to analyze the utility of a novel pregnancy options counseling curriculum by examining preclinical medical student perspectives on professional identity development as elicited by the curriculum.

## Materials and methods

The study took place at Indiana University School of Medicine (IUSOM). Second-year medical students enrolled in the Endocrine, Reproductive, Musculoskeletal, and Dermatologic (ERMD) course were eligible to participate in the study. The pregnancy options counseling curriculum was integrated into this required second-year course, which is the only IUSOM course to include dedicated reproductive health content at this stage of training; therefore, only second-year students were enrolled in the curriculum and eligible for inclusion. This study was determined to be exempt by the IUSOM institutional review board (Protocol # 18010).

### Curriculum

Our pregnancy counseling curriculum consisted of asynchronous didactic content on abortion, adoption, and pregnancy continuation (Supplemental material 2,3,4) followed by a live, interactive panel discussion. The mandatory 90-minute panel session was conducted via Zoom (Zoom Video Communications, San Jose, CA) webinar, in which a panel of multidisciplinary health professionals guided students through a clinical case scenario and shared their experiences providing counseling for pregnant patients. Panelists included 2 abortion care providers, an obstetrician/gynecologist who does not provide abortion care, an adolescent medicine specialist, and a licensed social worker who specializes in adoption.

The panel discussion focused on a clinical case presented via PowerPoint about a 25-year-old female presenting with an incidental positive pregnancy test (Supplemental material 1). Discussion questions aimed to explore how panelists navigated informing patients of a positive pregnancy test, counseling patients about their pregnancy options, and educating patients on how gestational age, past medical history, and socio-economic status may influence their decision. Students were able to interact with physician panelists through an anonymous question-and-answer chat function on Zoom. Students’ real-time questions/comments were not included in the analysis.

### Data collection

After the panel, students submitted a written reflection as part of their graded coursework, with the option to opt out of participation in the research study. Before submitting their reflections, students were informed that their responses would be included in a research study; submissions were graded for completion only and not for content. Learners were prompted to assess how their professional and personal identities align or conflict with pregnancy options counseling (Table 3). Specifically, students were asked: (1) “How do your personal and professional identities align or conflict with pregnancy options counseling?” and (2) “How do your personal beliefs regarding pregnancy options counseling impact your professional identity as a future physician?” Student reflections were de-identified before analysis.

The demographic characteristics of the participants were collected from IUSOM’s Data and Survey Vetting Committee

### Qualitative analysis

The de-identified responses were coded using the mixed-method analytic software, Dedoose (version 9.0.90).^7^ For prespecified analyses, a preliminary grid of codes was generated using the AAMC Physician Competencies for Professionalism: “Compassion, integrity and respect,” “Accountability,” “Autonomy,” “Patient needs,” “Sensitivity,” and “Commitment to ethical principles”.^1^^,^^6^ Three reviewers, an internal medicine physician, a second-year medical student, and a fourth-year medical student, coded all student responses independently and without knowledge of the other reviewers’ coding. The same code could be applied multiple times within one response if the response exhibited two or more distinct applications of the code. To support reviewer reflexivity, each coder documented their positionality before analysis. The internal medicine physician identified as a clinician with a commitment to evidence-based reproductive health care. The second-year and fourth-year medical student coders each identified as students navigating their own professional identity formation, with personal and clinical perspectives informed by their medical training at IUSOM. These positionalities were acknowledged throughout the coding process to minimize interpretive bias and support transparency in data analysis.

Reviewers met weekly for four weeks to discuss their thought processes, refine the coding scheme, and reach consensus on difficult or challenging passages. A fourth investigator, not otherwise involved in the coding process, reviewed discrepancies and determined final codes when reviewers were uncertain or faced disagreement. As common themes emerged, additional “Child codes” were created as sub-codes beneath the six AAMC professional competencies or “Parent codes” as illustrated in Table 2. These “Child codes” (ie, “providing referral” or “nonjudgmental care”) were added to the Professionalism Competency grid for more refined analysis. Using descriptive statistics in Dedoose, mean frequencies per reviewer of each professionalism competency were calculated.^8^

To assess the consistency of coding by all three reviewers, analysis of inter-reviewer reliability was conducted for each professional competency code.^9^ For simplicity, all Child codes were condensed to fall within their Parent code (Ie, “Patient-centered care” became “Patient Needs”), and an exploratory qualitative analysis was performed on fifteen excerpts per code to generate Cohen kappa coefficients (κ). κ was obtained using Dedoose, where agreement values of ≤0 indicated no agreement, 0.01–0.20 indicated none to slight, 0.21–0.40 indicated fair, 0.41–0.60 indicated moderate, 0.61–0.80 indicated substantial, and 0.81–1.00 indicated near perfect agreement.^10^

## Results

### Demographics

There were 352 student responses submitted on Canvas. Two students declined to participate in the study, and the reflections submitted by the two student authors of this study were also excluded. Final analysis included 348 responses, representing 94% of second-year students. Demographics of the second-year medical school class are shown in Table 1.

**Table 1:** Self-reported demographics of IUSOM class of 2025

Class of 2025	*N*=370 (%)
Sex	
Female	195 (52.7%)
Male	175 (47.3%)
Race	
White	189 (51.1%)
Asian	41 (11.1%)
Black or African American	31 (8.4%)
Two or more races	67 (18.1%)
Hispanic/Latino	23 (6.2%)
Unknown	16 (4.3%)
Ethnicity	
Hispanic or Latino	36 (9.7%)
Not Hispanic or Latino	334 (90.3%)
In state/out of state	
In state	310 (83.8%)
Out of state	60 (16.2%)

Brown. Pregnancy options counseling in medical education. J Obstet Gynecol 2026.

### Qualitative results overview

**Figure 2:**
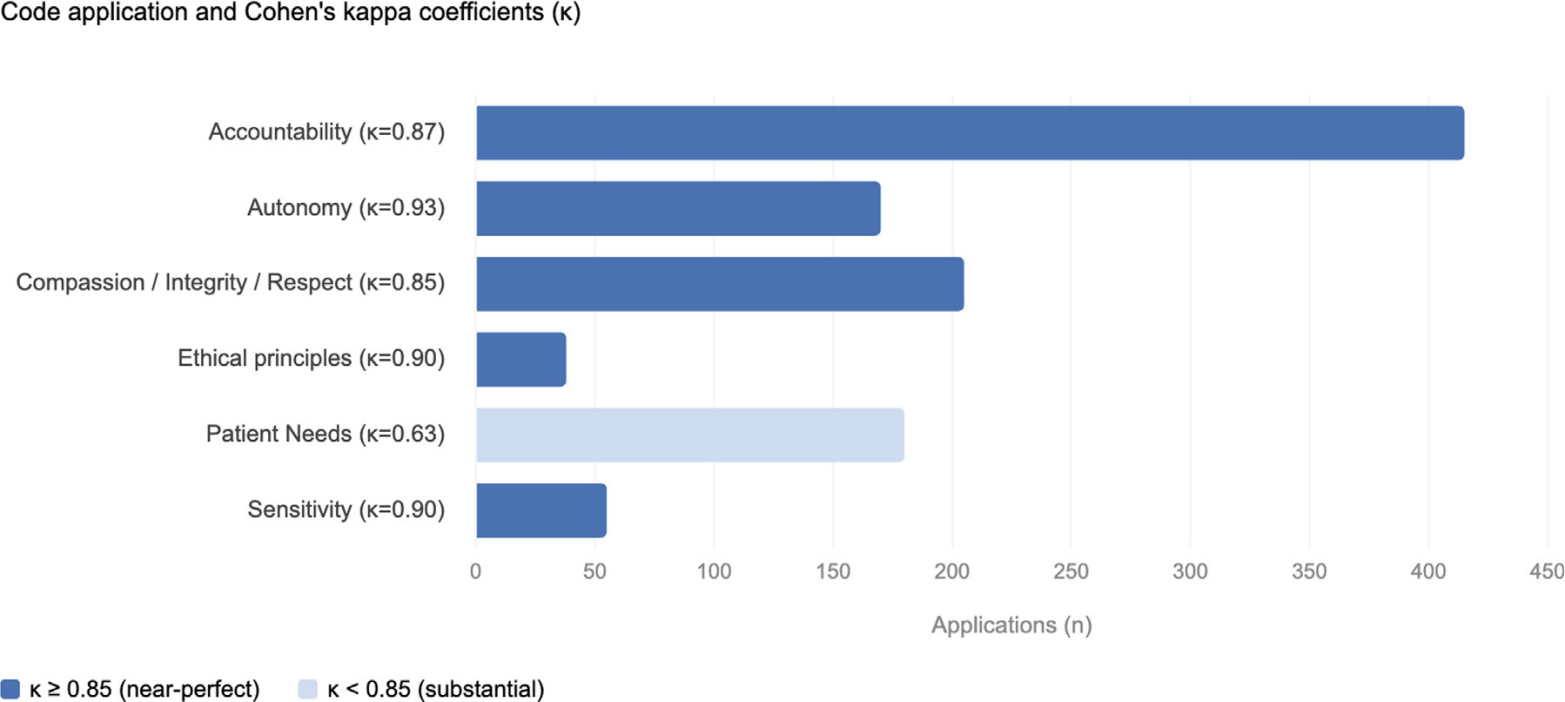
Code application and Cohen's kappa coefficients (K)

Professional competency codes, their application frequency, and κ are exhibited in Figure 2. The inter-reviewer reliability test showed significant agreement between reviewers: “Compassion, integrity and respect,” “Accountability,” “Autonomy,” “Sensitivity,” and “Commitment to ethical principles” were coded with near-perfect agreement (all κ≥0.85) while “Patient Needs” was found to have substantial agreement (κ=0.63). The most common competencies represented were “Accountability” (n=416), “Compassion, Integrity, Respect” (n=201), and “Patient Needs” (n=178). The AAMC competencies/”parent codes”, their exemplars/”child codes”, their mean frequencies per coder, and exemplary quote(s) are illustrated in Table 2.

Brown. Pregnancy options counseling in medical education. J Obstet Gynecol 2026.

**Table 2:** AAMC professionalism competencies with exemplary quotes

AAMC competency/“parent codes”	Exemplars/”child codes”	Mean frequency per reviewer *N=*	Exemplary quote(s)
Compassion, integrity, and respect for others (humanism)	• Nonjudgmental care • Respect for patients • Empathy for patients • Make patients comfortable	201	“I believe that everyone’s family and personal dynamics are so different and unique that in order to provide the best quality of care for patients, we as providers need to be a nonjudgmental party who helps them fully explore their options.”
Responsiveness to patient needs that supersedes self-interest (“patient needs superseding self-interest”)	• Understanding the patient’s needs / perspective • Desire to help the patient do the best thing for themselves	178	“Although I don’t personally support abortion, I recognize that it is my patient’s right to know what their legal options are even if that includes abortion. I would want my patients to have access to accurate and reliable information to make the best decision when it comes to their pregnancy.”
Respect for patient privacy and autonomy (“patient autonomy”)	• Patient-centered care that respects autonomy • Lack of bias when that influences counseling	166	“As a medical professional, I must respect the patient's autonomy and support their decisions while providing them with the best possible care.”
Accountability to patients, society, and the profession (“physician accountability”)	• Providing all options • Informed decision making, tools for informed decision making • Providing referral • Advocating for patient autonomy • Be the best possible physician for patients • Assist patient in reaching their goals	416	“While it is important for a physician to maintain their own morals and not compromise their beliefs to serve the patient, it's necessary to provide the patient with information on all options. For example, a physician may not be morally inclined to perform an abortion, but it is still their duty to provide information on abortions to the patient and provide a referral to an abortion specialist if necessary.”
Sensitivity and responsiveness to a diverse patient population (“sensitivity to diverse populations”)	• Advocating for equity in healthcare and for marginalized groups	54	“This [education on counseling] shifts my identify as a healthcare provider from that of a person who provides a service and cares for a patient to that of a person of privilege and education who has a responsibility to advocate for vulnerable populations, seeking out new solutions to these roadblocks as they arise.”
Commitment to ethical principles (“commitment to ethical principles”)	• Ethical principles	38	“My personal values and religious beliefs make me morally opposed to abortion, which, on the surface, conflicts with my future ability to counsel on pregnancy options. However, I know that I have the ethical responsibility to inform my patients about their pregnancy options, even if abortion is a service that I cannot provide. Sometimes it is difficult for me to reconcile these 2 ethical obligations, but whenever I do struggle, I make myself remember that my rights end where the patient’s begin.” “My goal as a future physician is to be able to put my patient first. Unfortunately, laws will dictate the way that I practice and the resources that I can provide my patients.”

Brown. Pregnancy options counseling in medical education. J Obstet Gynecol 2026.

**Table 3:** Written reflection prompt

When we become physicians, we must incorporate the values of the medical profession with our personal values, including times when those values differ. For example, you might not want life-prolonging procedures such as tube feeding for yourself if you had advanced dementia, but as a physician you must counsel patients and families about all the nutrition support options for patients with this condition. Patients may choose an option that you would not choose for yourself. Professional experiences can shift our concept of ourselves. The 2 questions below ask you to reflect on your self-concept through what you bring to a situation and what the situation brings to you. How do your personal identity, beliefs, and values and your professional identity as a future physician align/conflict in your counseling on pregnancy options? How does your counseling on pregnancy options impact your concept of yourself (your personal identity, beliefs, and values) and your professional identity as a future physician?

Brown. Pregnancy options counseling in medical education. J Obstet Gynecol 2026.

### Competency 1: Compassion, integrity, and respect for others (humanism)

Competency 1 was identified an average of 201 times per coder (κ≥0.85). Students consistently exhibited empathy and compassion for patients by wanting to understand the patients” goals, emotions, and values involved in their decision-making process. Even if learners did not personally agree with abortion, nearly all students put this difference aside to support the patient and exhibit empathy.

### Competency 2: Responsiveness to patient needs that supersedes self-interest (“patient needs superseding self-interest”)

Competency 2 was identified an average of 178 times per coder (κ≥0.63). Most students articulated a duty to prioritize patient needs above self-interest, with the majority expressing commitment to unbiased care. A minority described internal conflict when personal or moral beliefs conflicted with the patient’s decision.

### Competency 3: Respect for patient privacy and autonomy (“patient autonomy”)

Competency 3 was identified an average of 166 times per coder (κ≥0.93), with students frequently citing the importance of autonomy and patient privacy.

### Competency 4: Accountability to patients, society, and the profession (“physician accountability”)

Competency 4 was identified an average of 416 times per coder (κ≥ 0.85). Competency 4 was coded the most frequently. Several reflections demonstrated accountability to patients by expressing commitment to providing comprehensive counseling on all pregnancy options, a desire to provide tools for informed decision making, and providing referrals when appropriate. Students tended to agree that it was the physician’s responsibility to present all options regardless of the physician’s personal beliefs on pregnancy termination. Students wrote that they wished to facilitate solutions to their patients’ needs while being the best physician possible for their patients. Learners expressed a prominent level of awareness of their own biases and how those personal biases could affect their professional obligation to counsel patients.

### Competency 5: Sensitivity and responsiveness to a diverse patient population

Competency 5 was identified an average of 54 times per coder (κ≥ 0.90). Students exhibited this competency by advocating for marginalized groups and articulating the effect of social determinants of health on pregnancy outcomes and the accessibility of comprehensive pregnancy options counseling.

### Competency 6: Commitment to ethical principles

Competency 6 was the least frequently coded, averaging 38 per reviewer (κ≥ 0.90). Responses referenced the volatile political climate, legislative interference, and restrictions on reproductive healthcare as factors impacting physicians’ ability to honor medical ethics. Some students demonstrated alignment between personal and professional values; others struggled to reconcile personal moral beliefs with the pillars of medical ethics.

### Other themes

Other common themes noted in the student reflections outside of our coding scheme included the influence of the students’ upbringing, religion, ethical views, and political views as they inform their professional identity. Additionally, several students explicitly expressed a desire for expanded education on abortion care, requesting more clinical detail on abortion procedures, legal frameworks, and access pathways. Representative responses included requests for “more information on how to counsel patients on all options without bias” and expressions of wanting to “learn more about what options are actually available to patients in Indiana.” Several student reflections discussed how their upbringing in rural Indiana, mentioning that being raised in a “small, conservative town” greatly influenced their initial views of abortion. The IUSOM class of 2025 consists mainly of in-state students (86.6%). While many of the students who contextualized their responses within geographical or cultural influences expressed personal discomfort with abortion care, several expressed evolving comfort with their role in options counseling after the panel, including referring patients to an abortion provider.

## Comment

### Principal findings

The pregnancy options counseling curriculum demonstrated utility in professional identity development among second-year medical students as outlined by the AAMC professionalism competencies. The curriculum emphasized the importance of using neutral language when diagnosing a patient with pregnancy, presenting the patient with all options, including those that may conflict with personal beliefs, and the importance of referring patients to another provider if the physician does not feel confident in their ability to counsel in an unbiased manner. The case challenged students to reconcile their personal beliefs and their professional obligations, and most student responses were sincere, thoughtful, and compassionate. There was an overall sense of appreciation for the curriculum, and learners valued the representation of diverse perspectives in the panel discussion. It is important to acknowledge, however, that professionalism itself is not a static or politically neutral construct. The AAMC competencies provide a shared framework, but their application in reproductive health care is increasingly complicated by state laws that may prohibit or restrict abortion counseling, referral, or even discussion. In such environments, what constitutes “professional” behavior may be legally contested, and educators must prepare students to navigate the tension between professional ethical obligations and evolving legal constraints. This curriculum represents one approach to that preparation, but its applicability will vary depending on institutional and jurisdictional context.

### Results in the context of what is known

The overall positive response to the panel is consistent with prior literature, which revealed a strong interest by students for expanded education on abortion care.^11^ Our pregnancy options counseling curriculum accounts for students’ educational desires, facilitates standardized professionalism development, and fills a well-documented gap in undergraduate medical education.^12^ A prior study similarly found that OB/GYN ethical cases aided professionalism development and allowed students to explore professional duty, communication, and ethical values applicable across specialties.^13^ As access to abortion continues to become more restricted, foundational, comprehensive education about pregnancy options counseling is of the utmost importance, regardless of specialty interest.

### Clinical implications

A standardized curriculum on pregnancy options counseling will benefit patients by improving access to unbiased, evidence-based information, addressing misinformation about abortion, and decreasing stigma. Medical schools adopting this curriculum can expect to train physicians better equipped to provide patient-centered reproductive counseling across diverse practice environments.

### Research implications

Future research should employ anonymous or nongraded reflection formats to reduce social desirability bias and more accurately capture authentic student attitudes toward pregnancy options counseling. Longitudinal assessment incorporating objective structured clinical encounters (OSCEs) with standardized patients would provide a more comprehensive evaluation of how curriculum exposure translates into clinical behavior. Additionally, multi-institutional studies across varied legal and political environments would strengthen generalizability and clarify how state-level restrictions on abortion care affect the feasibility and impact of options counseling curricula. Expanding the qualitative coding scheme to capture themes around legal literacy, institutional constraints, and conscience rights would address gaps identified in the current analysis.

### Strengths and limitations

Our study strengths include the large sample size and high participation rate, resulting in meaningful, diverse responses that accounted for a broad range of attitudes and perspectives surrounding pregnancy options. As the largest medical school in the country, with a predominantly in-state student body drawn from a politically and geographically diverse state, this study captures a broad range of perspectives on pregnancy options counseling from students whose personal and community values vary considerably.

A significant limitation of this study is the potential for social desirability bias. Student reflections were submitted as graded coursework and were not anonymous at the time of submission, being de-identified only after submission for research analysis. Although grading was based solely on completion and not on content, the graded, nonanonymous context of submission may have encouraged students to align their written responses with perceived professional or institutional expectations rather than authentic personal perspectives. Students who held beliefs discordant with evidence-based pregnancy options counseling may have been particularly hesitant to express those views, potentially overstating the degree of alignment between personal and professional values in our sample. This limits our ability to draw strong conclusions about the extent to which the curriculum genuinely shifted professional identity formation versus eliciting socially desirable responses. Future studies should consider anonymous reflection formats or validated instruments to more accurately capture authentic student perspectives.

### Sociopolitical and legal context of abortion education

The generalizability of this curriculum is substantially shaped by the sociopolitical and legal landscape of abortion care in the United States. Following the 2022 Supreme Court decision in *Dobbs v. Jackson Women’s Health Organization*, numerous states have enacted laws that restrict or criminalize abortion care, referral, and in some jurisdictions, even the provision of information about abortion options. These restrictions directly implicate medical education: in states where discussing or referring for abortion is legally prohibited, the ethical obligation to provide comprehensive pregnancy options counseling may conflict with state law. This raises critical questions about the feasibility, ethics, and legal risk of implementing this curriculum beyond institutions in states where abortion remains legal and accessible. Medical educators and institutions must grapple with the responsibility to train physicians who can provide evidence-based care while also navigating legal environments that may penalize such care. The curriculum was developed and implemented in Indiana, a state that enacted a near-total abortion ban following *Dobbs*. The continued delivery of this curriculum in such an environment reflects institutional commitment to comprehensive reproductive health education, but also highlights the challenges educators and students face in engaging with this content in legally restrictive contexts. Institutions operating under restrictive abortion laws should consider the legal and ethical implications of implementing this curriculum and may need to adapt content accordingly — for example, focusing on values clarification, harm-reduction language, and legal literacy without explicitly providing referral training where prohibited.

Another important limitation is that the implementation of pregnancy options counseling will likely not occur in a politically or legally neutral context. Abortion care has always been one of the most contested topics in medicine, and as a result, professional standards surrounding pregnancy options counseling are constantly being shaped by ongoing political and legal pressures. As abortion laws become more restrictive, future physicians may not be able to provide comprehensive options counseling as institutions are preemptively curtailing abortion practices to avoid perceived legal or reputational risks. These institutional and legal dynamics interfere with the ethical and professional duties of providers to provide all options, while raising questions of access, equity, and professional responsibility in preparing future physicians to provide patient-centered care.

In response to these challenges, curricular strategies will need to be adapted for use in legally restrictive environments while still fostering core professionalism competencies. For instance, training in harm reduction language can equip students with strategies to support patients in ways that reduce stigma while emphasizing safety. Additionally, more curriculum is needed to emphasize legal literacy, conscience rights, and professional ethics to help prepare students to navigate complex practice environments where institutional policy and law negatively intersect with patient care. These additions to the curriculum will help ensure that medical students develop skills central to respectful, nonjudgmental care in the face of legal and institutional barriers.

While the utility of written reflection to evaluate knowledge and attitudes surrounding professional identity development is substantial, behavior is the true test of change.^14^, ^15^ Students’ actions may vary from their attitudes in different social, political, and medical contexts.^12^ In future evaluations, objective structured clinical encounters (OSCEs) using standardized patients may yield a more holistic assessment of students’ knowledge, attitudes, and behaviors towards pregnancy options counseling. Lastly, there were themes in student responses that were unaccounted for by our coding scheme, which could provide additional insight. Subsequent qualitative research on this topic could expand the coding scheme to better account for students’ thoughts and insights.

## Conclusion

Pregnancy options counseling is an effective vehicle for professional identity development, urging students to reconcile personal values with professional obligations. This curriculum was particularly effective in eliciting reflections on physician accountability, patient autonomy, and compassion. We encourage other medical schools to adopt this curriculum to address gaps in reproductive health education and promote professionalism.

## Declaration of generative AI and AI-assisted technologies in the manuscript preparation process

*During the preparation of this work the author(s) used Claude AI in order to proofread and suggest edits for final submission. After using this tool/service, the author(s) reviewed and edited the content as needed and take(s) full responsibility for the content of the published article.*

## Tweetable statement

The addition of a pregnancy options counseling curriculum promotes the development of professionalism in preclinical medical students.

## Ethical approval statement

All procedures were performed in compliance with relevant laws and institutional guidelines and have been approved by the appropriate institutional committee: Indiana University School of Medicine Institutional Review Board - Protocol #18010.

## CRediT authorship contribution statement

**Lucy D Brown:** Conceptualization, Formal analysis, Investigation, Methodology, Writing – original draft, Writing – review & editing. **Carli A King:** Conceptualization, Data curation, Formal analysis, Investigation, Writing – original draft, Writing – review & editing. **Leah J Peipert:** Conceptualization, Data curation, Methodology. **Jeffrey F Peipert:** Supervision. **Julianne Stout:** Conceptualization, Data curation, Investigation, Methodology. **Amy Caldwell:** Conceptualization, Funding acquisition, Supervision.

## Appendix Supplementary materials

Image, application 1

Image, application 2

Image, application 3

Image, application 4

## Footnotes

Supplementary material associated with this article can be found in the online version at doi:10.1016/j.xagr.2026.100656.
