# Supplementary material for: Pregnancy options counseling in medical education and professionalism development
Source: AJOG Glob Rep. 2026 May 19;6(3):100656. doi: 10.1016/j.xagr.2026.100656 (PMC13314970; doi:10.1016/j.xagr.2026.100656)
Supplement: Supplementary file 1 [file mmc1.zip › mmc1.pptx]

## Slide 1
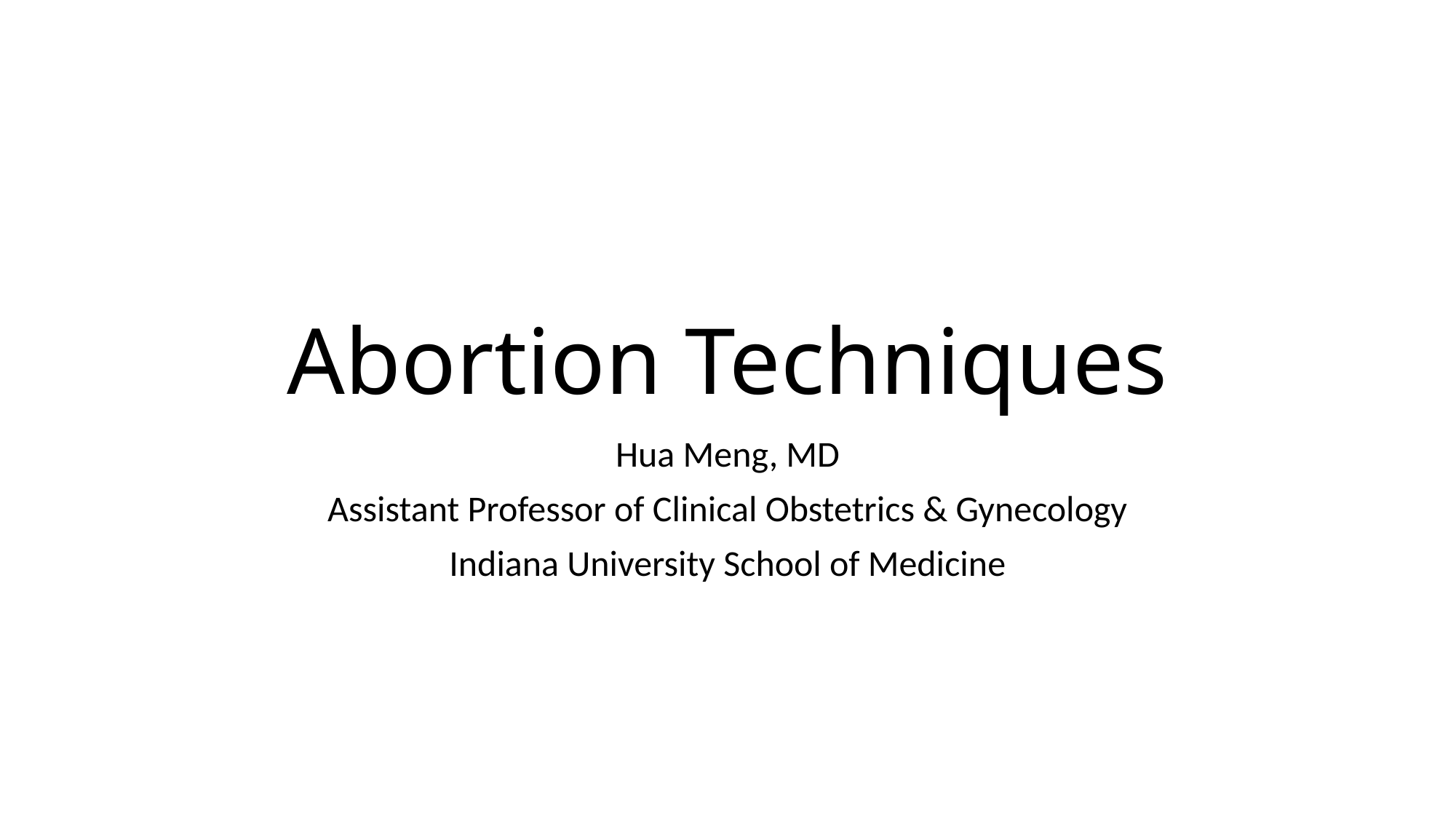

# Abortion Techniques
Hua Meng, MD
Assistant Professor of Clinical Obstetrics & Gynecology
Indiana University School of Medicine

## Slide 2
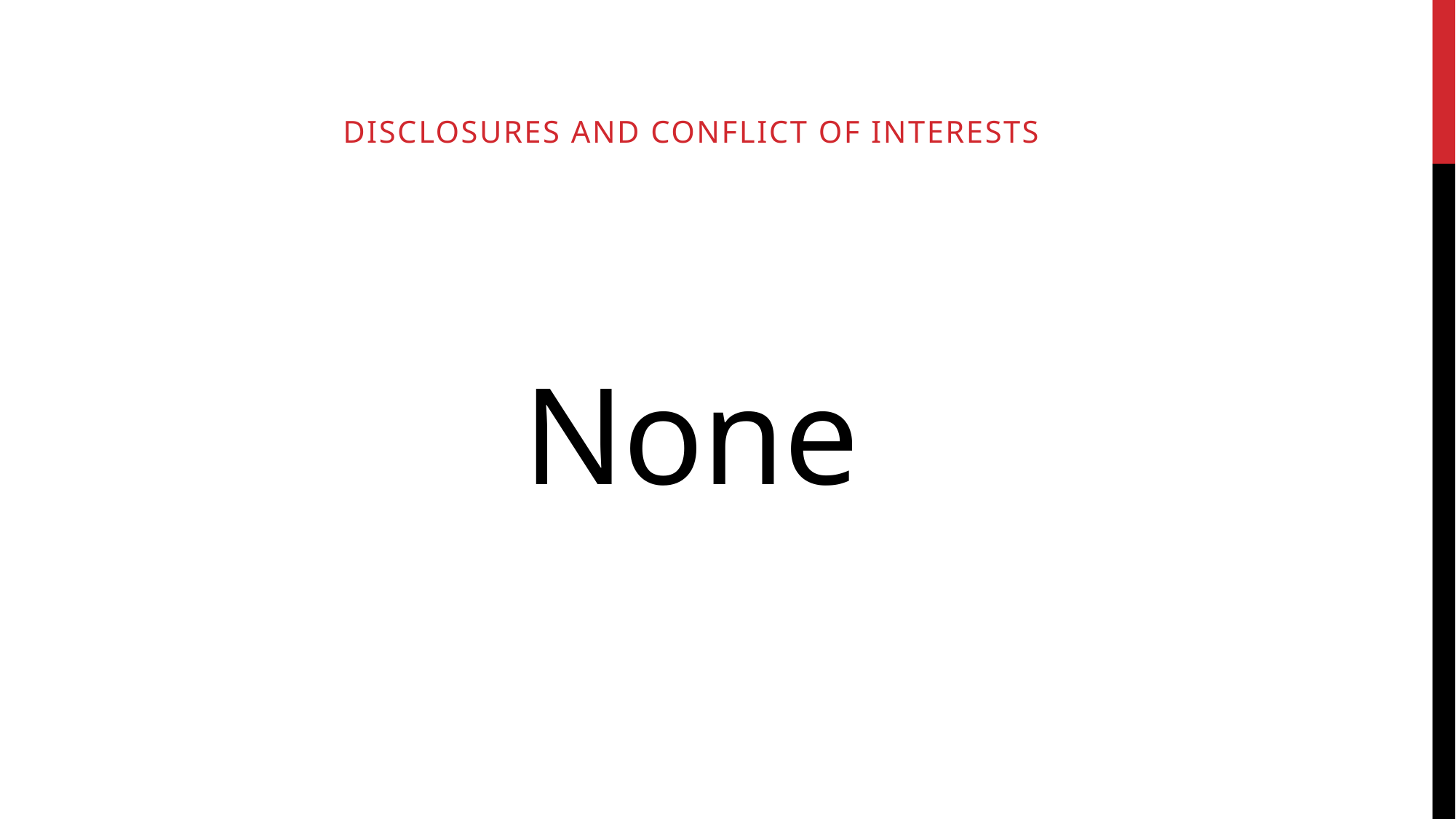

DISCLOSURES AND CONFLICT OF INTERESTS
# None

## Slide 3
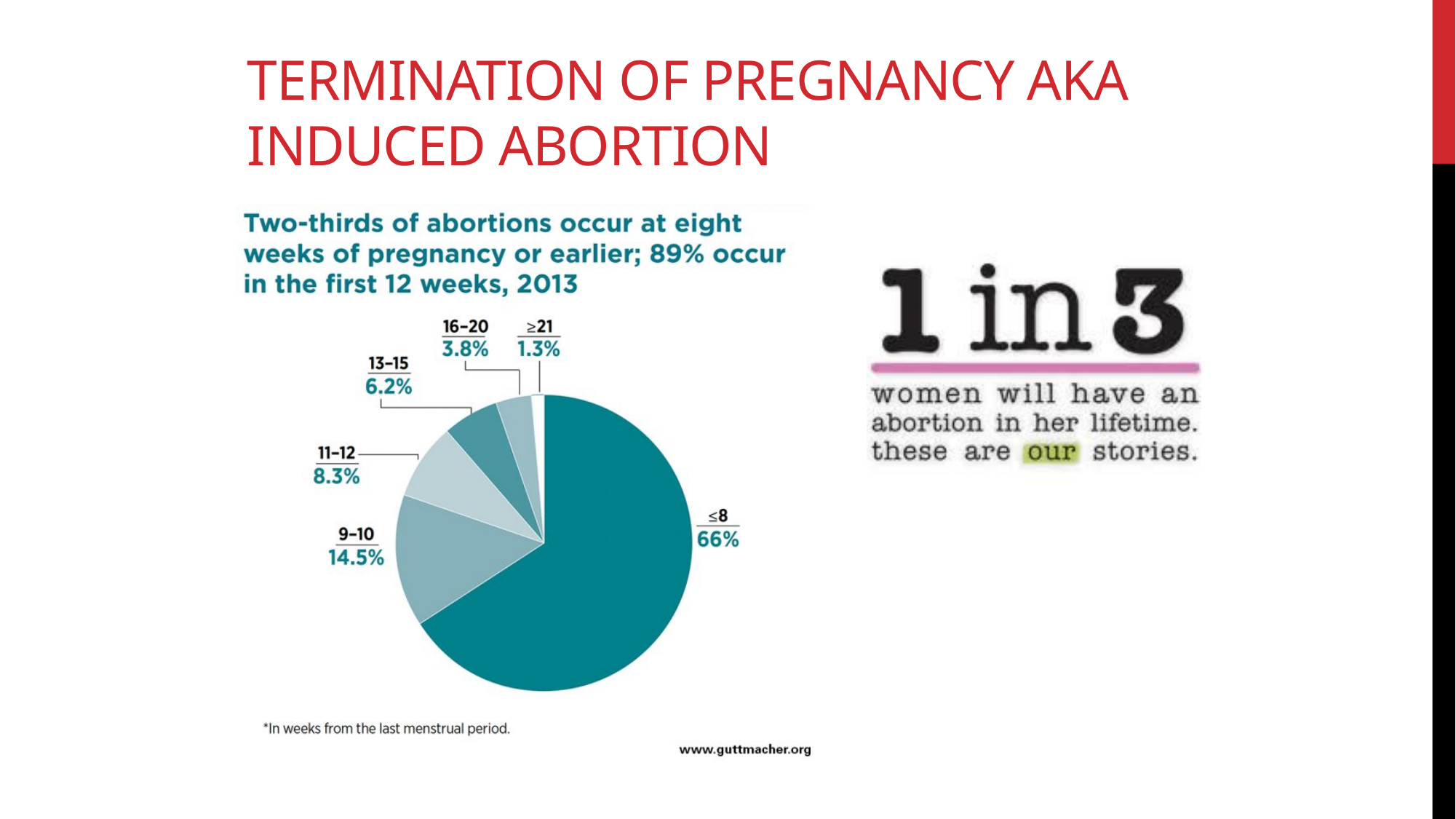

# Termination of pregnancy aka induced abortion

## Slide 4
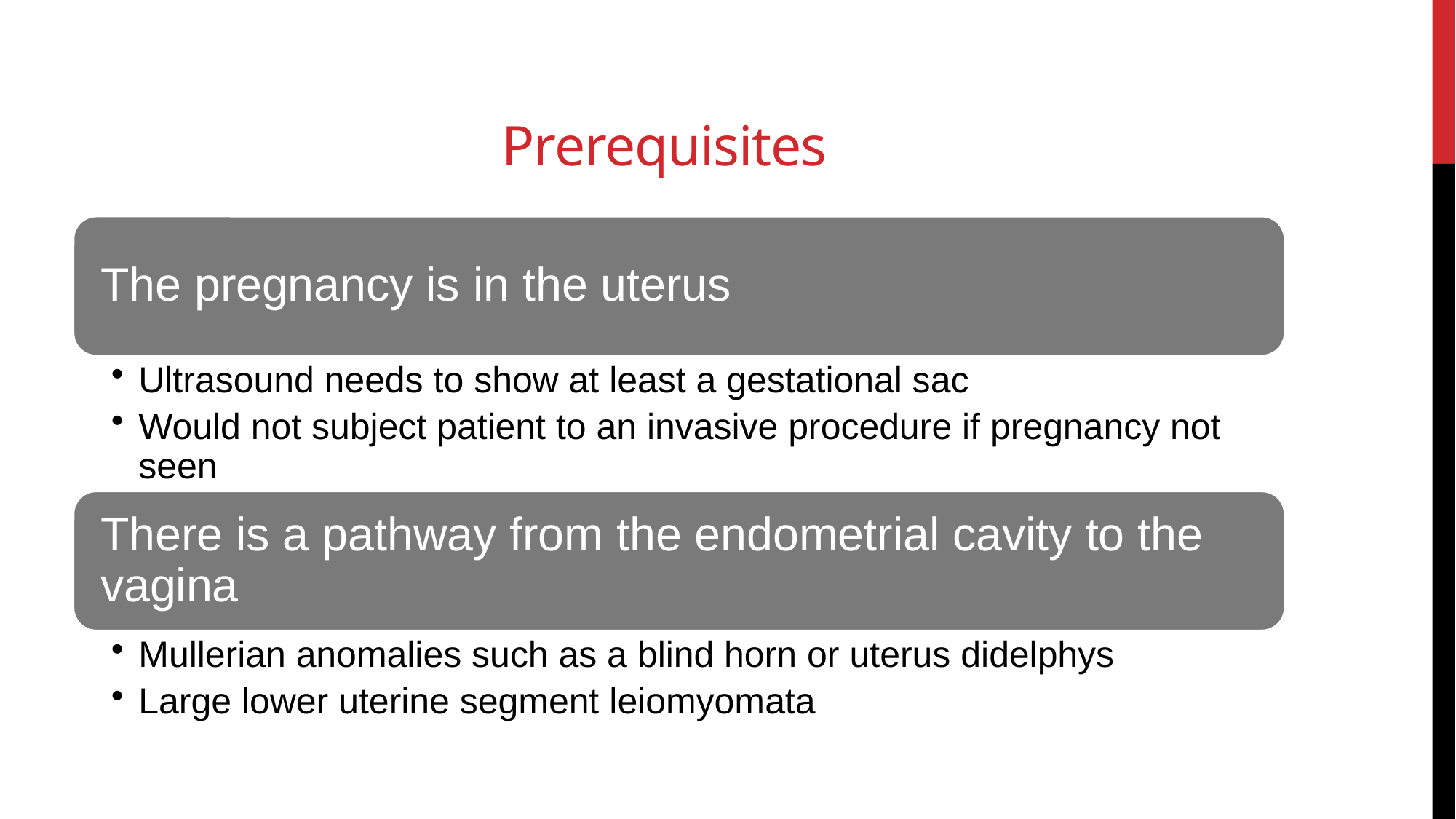

# Prerequisites

## Slide 5
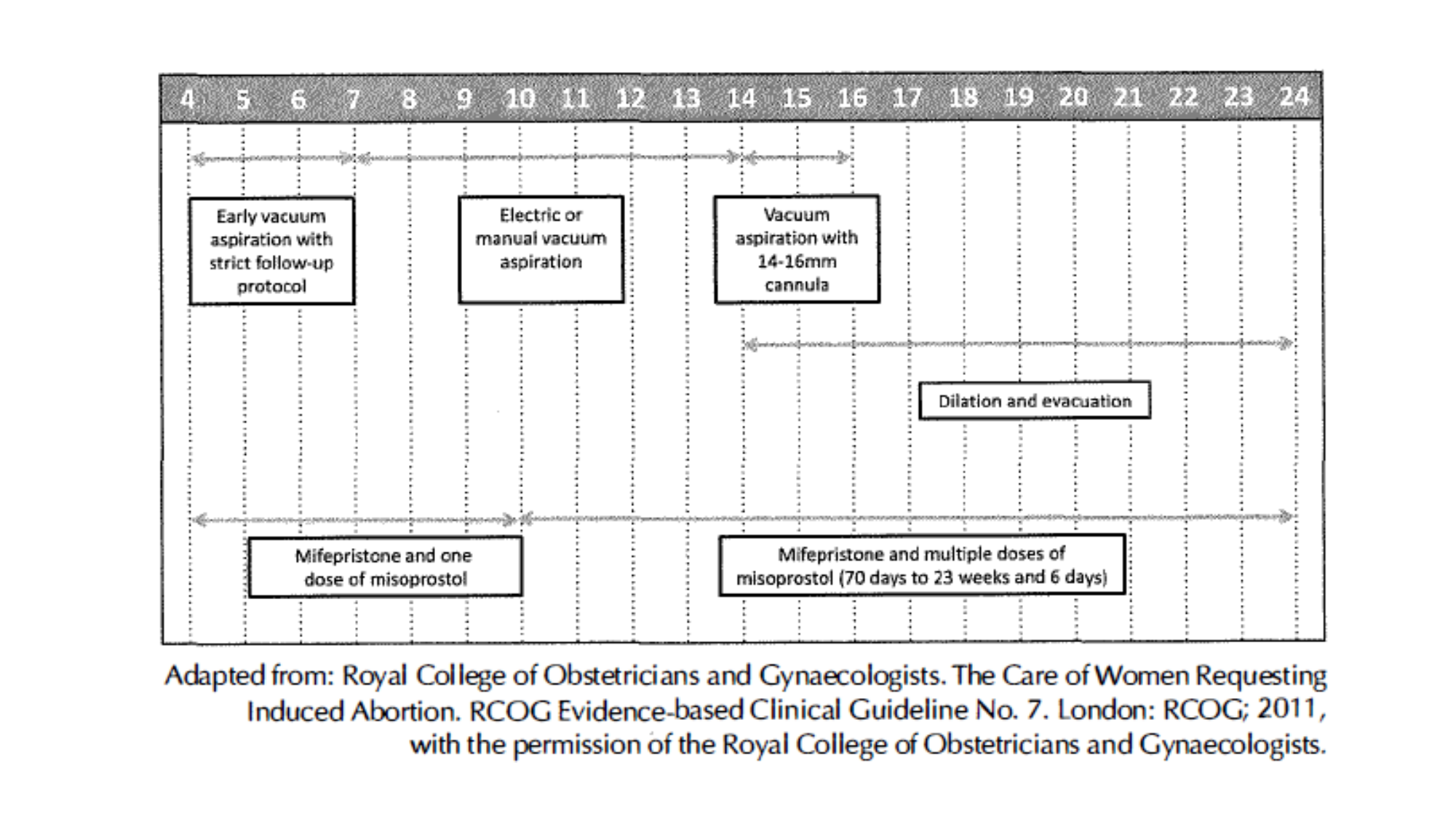

## Slide 6
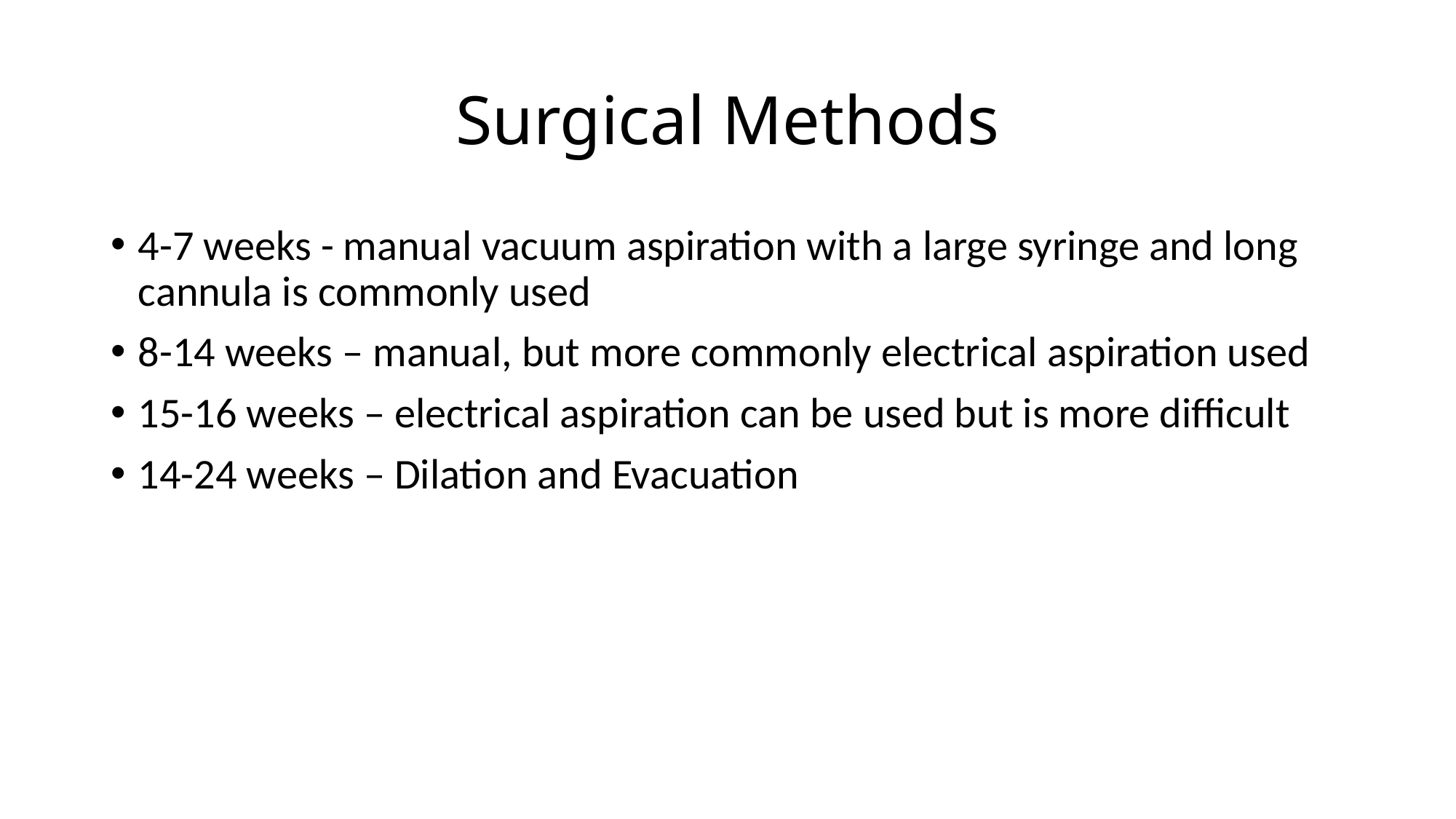

# Surgical Methods
4-7 weeks - manual vacuum aspiration with a large syringe and long cannula is commonly used
8-14 weeks – manual, but more commonly electrical aspiration used
15-16 weeks – electrical aspiration can be used but is more difficult
14-24 weeks – Dilation and Evacuation

## Slide 7
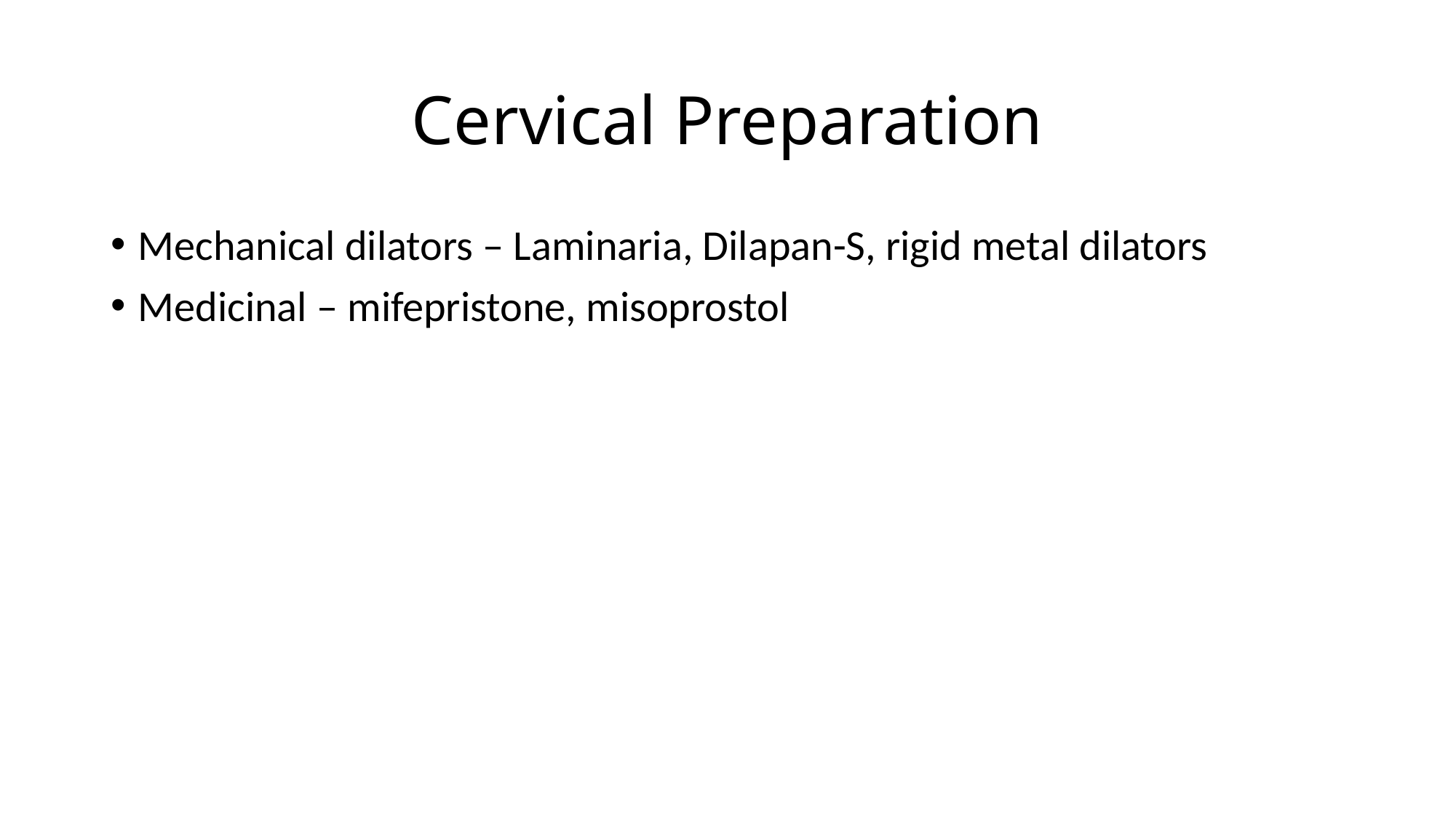

# Cervical Preparation
Mechanical dilators – Laminaria, Dilapan-S, rigid metal dilators
Medicinal – mifepristone, misoprostol

## Slide 8
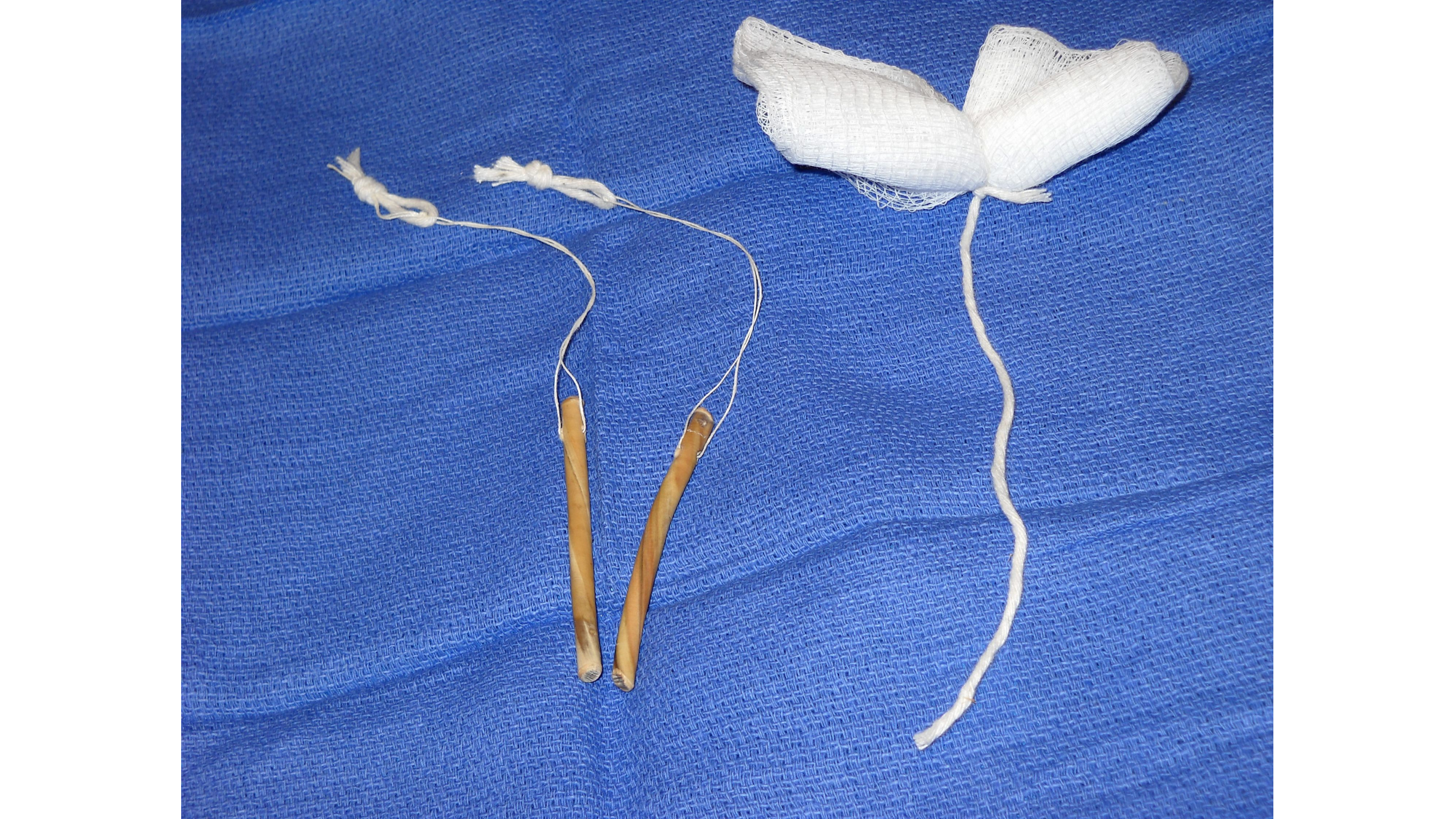

## Slide 9
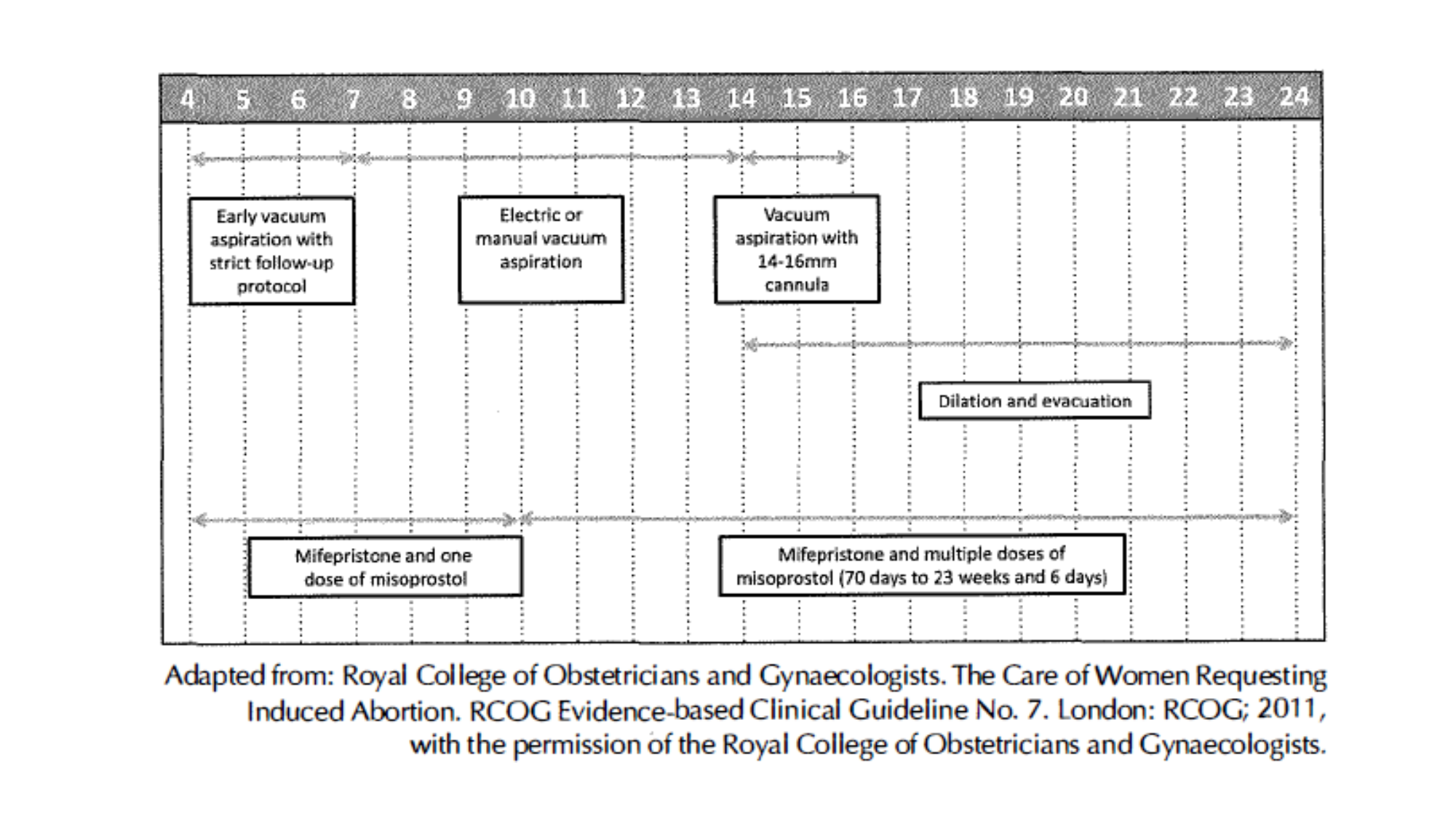

## Slide 10
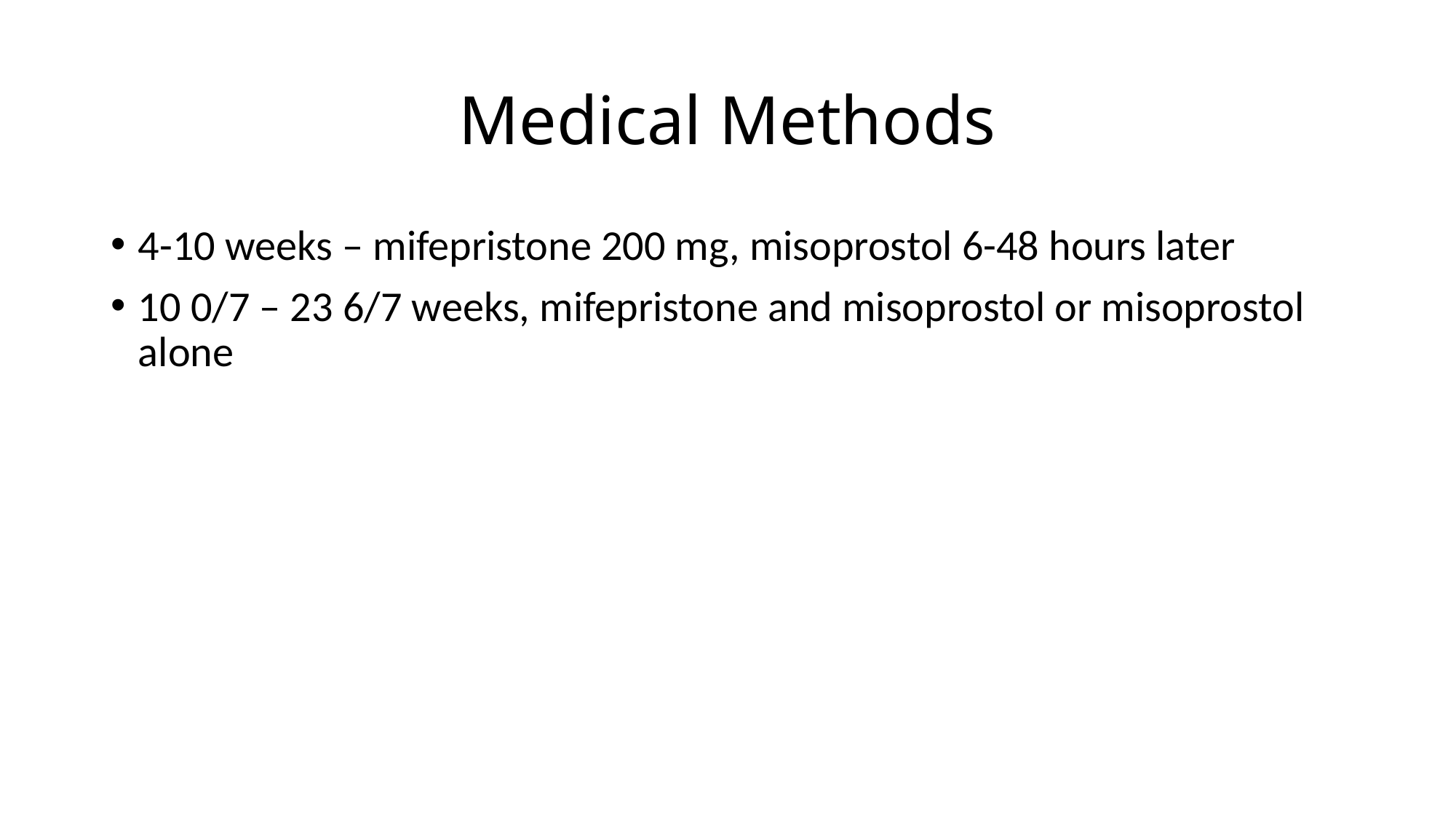

# Medical Methods
4-10 weeks – mifepristone 200 mg, misoprostol 6-48 hours later
10 0/7 – 23 6/7 weeks, mifepristone and misoprostol or misoprostol alone

## Slide 11
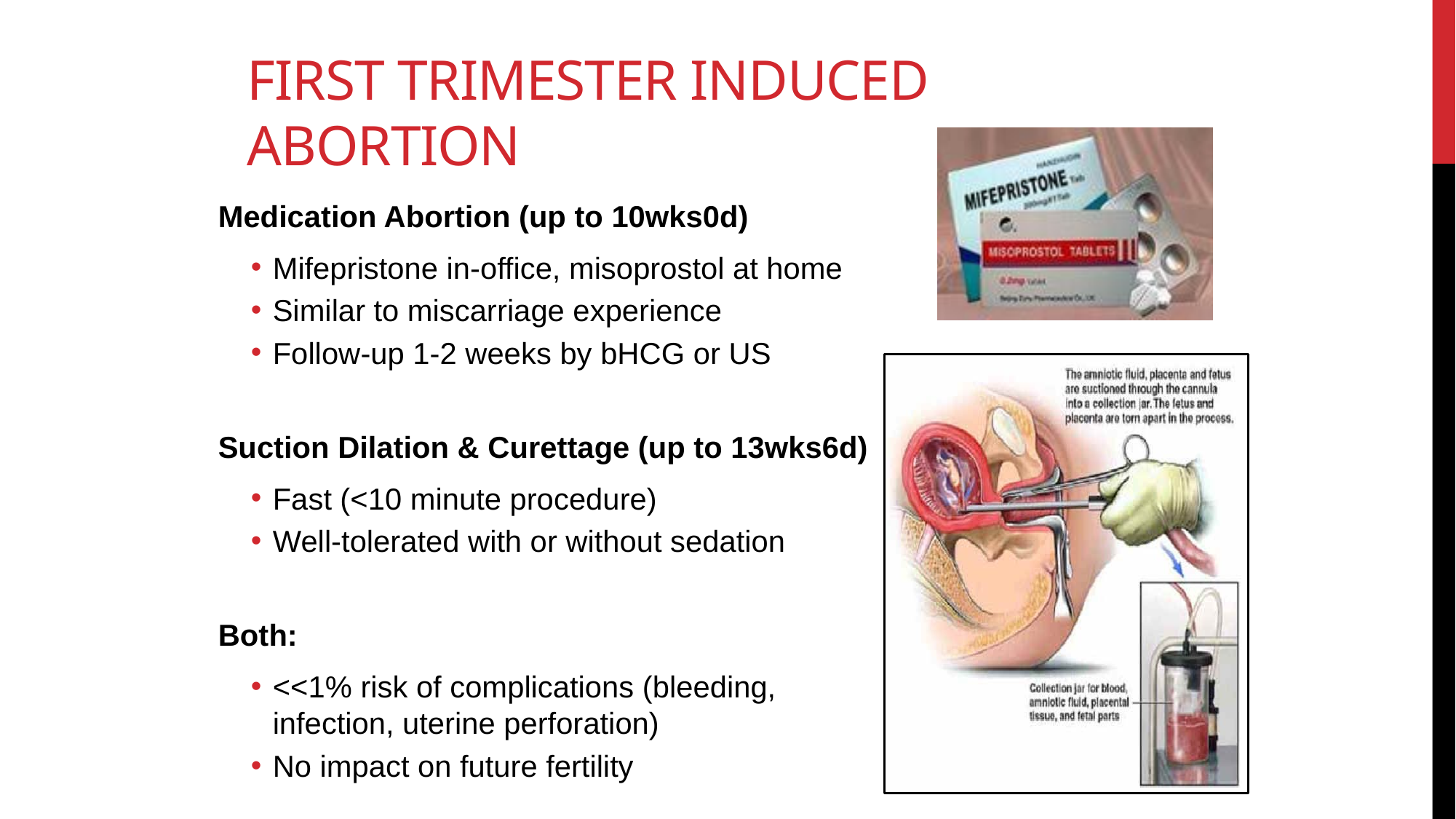

# First trimester induced abortion
Medication Abortion (up to 10wks0d)
Mifepristone in-office, misoprostol at home
Similar to miscarriage experience
Follow-up 1-2 weeks by bHCG or US
Suction Dilation & Curettage (up to 13wks6d)
Fast (<10 minute procedure)
Well-tolerated with or without sedation
Both:
<<1% risk of complications (bleeding, infection, uterine perforation)
No impact on future fertility

## Slide 12
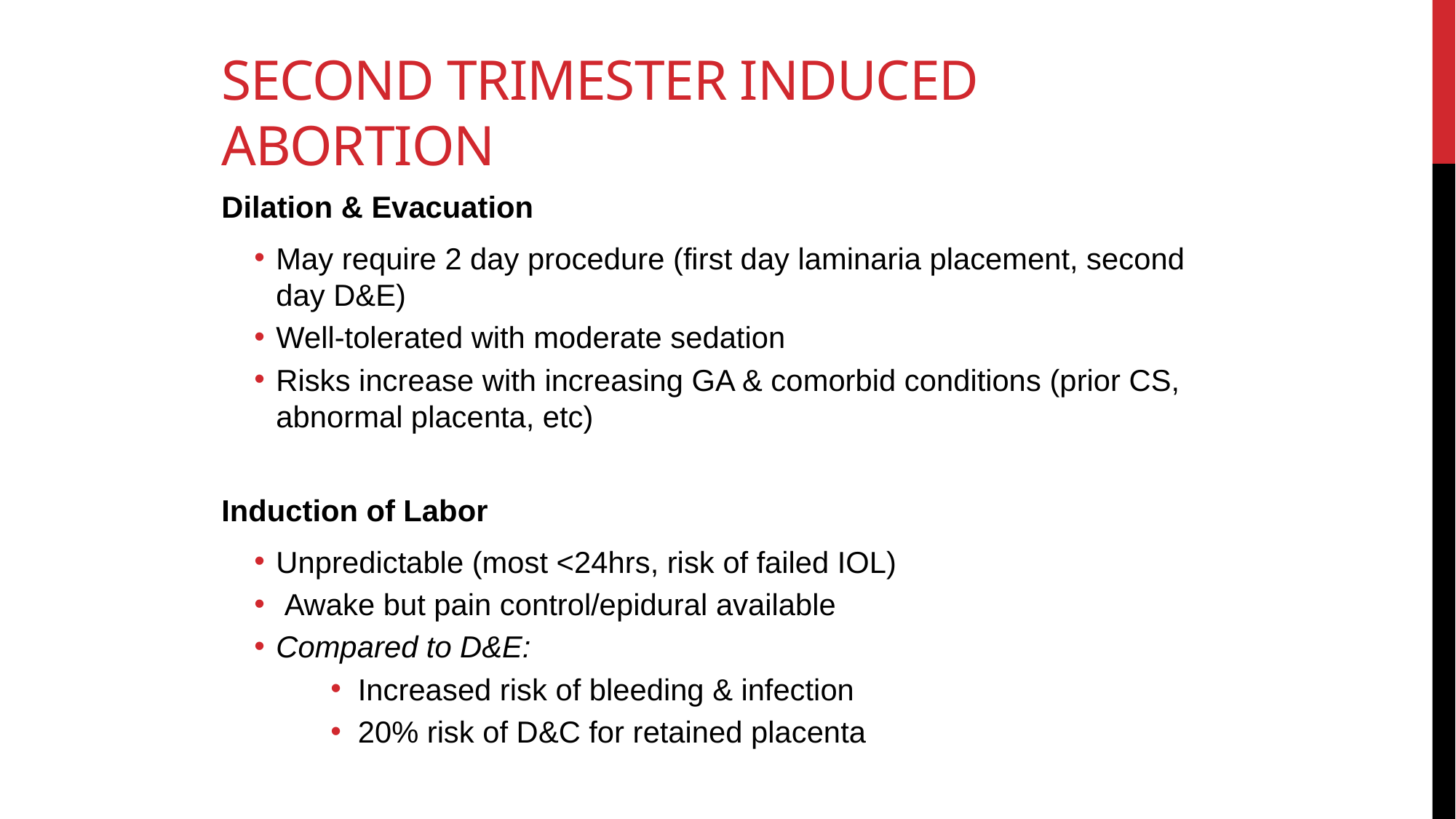

# Second trimester induced abortion
Dilation & Evacuation
May require 2 day procedure (first day laminaria placement, second day D&E)
Well-tolerated with moderate sedation
Risks increase with increasing GA & comorbid conditions (prior CS, abnormal placenta, etc)
Induction of Labor
Unpredictable (most <24hrs, risk of failed IOL)
 Awake but pain control/epidural available
Compared to D&E:
Increased risk of bleeding & infection
20% risk of D&C for retained placenta

## Slide 13
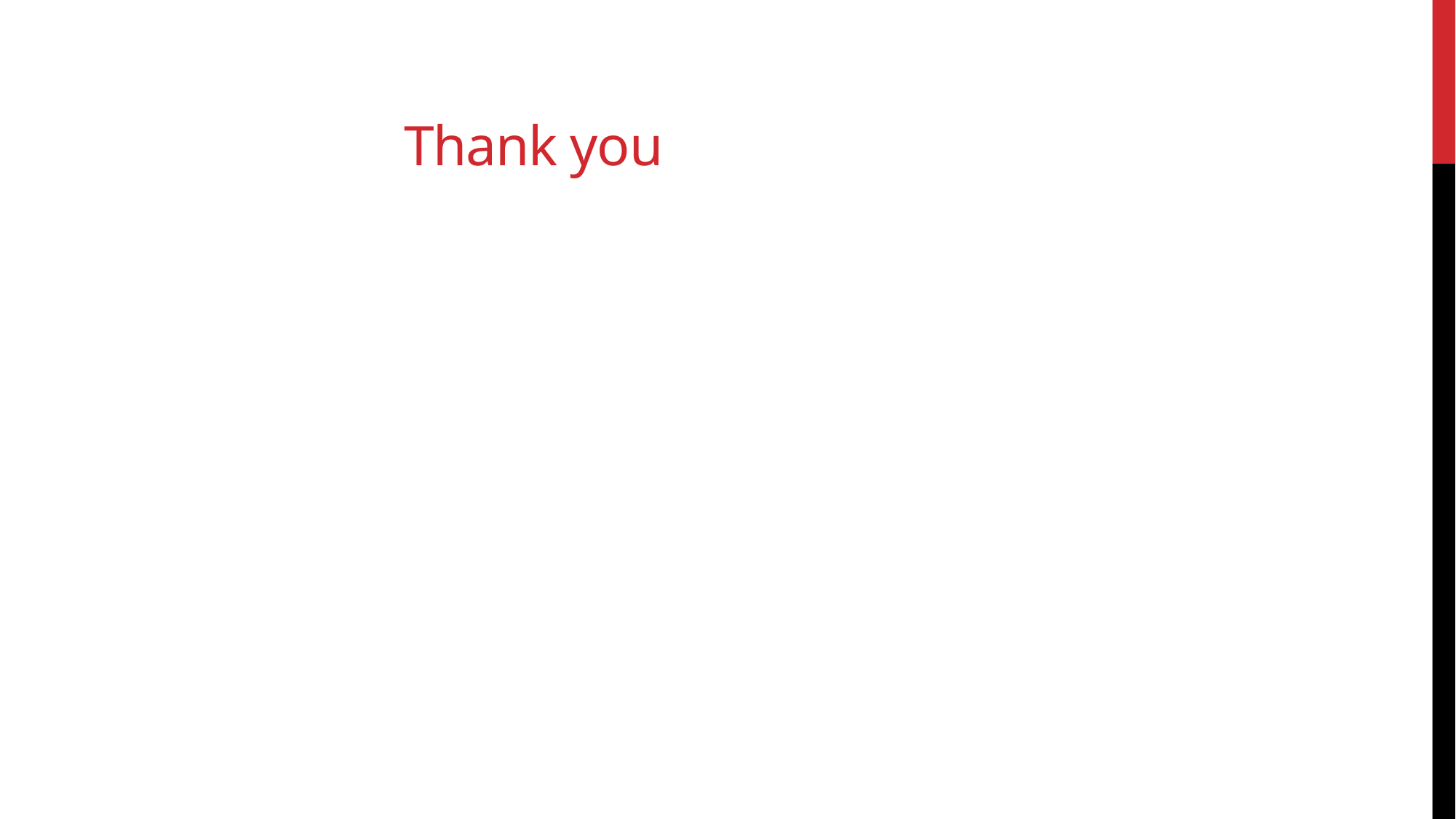

# Thank you
