# Supplementary material for: Pregnancy options counseling in medical education and professionalism development
Source: AJOG Glob Rep. 2026 May 19;6(3):100656. doi: 10.1016/j.xagr.2026.100656 (PMC13314970; doi:10.1016/j.xagr.2026.100656)
Supplement: Supplementary file 2 [file mmc2.zip › mmc2.pptx]

## Slide 1
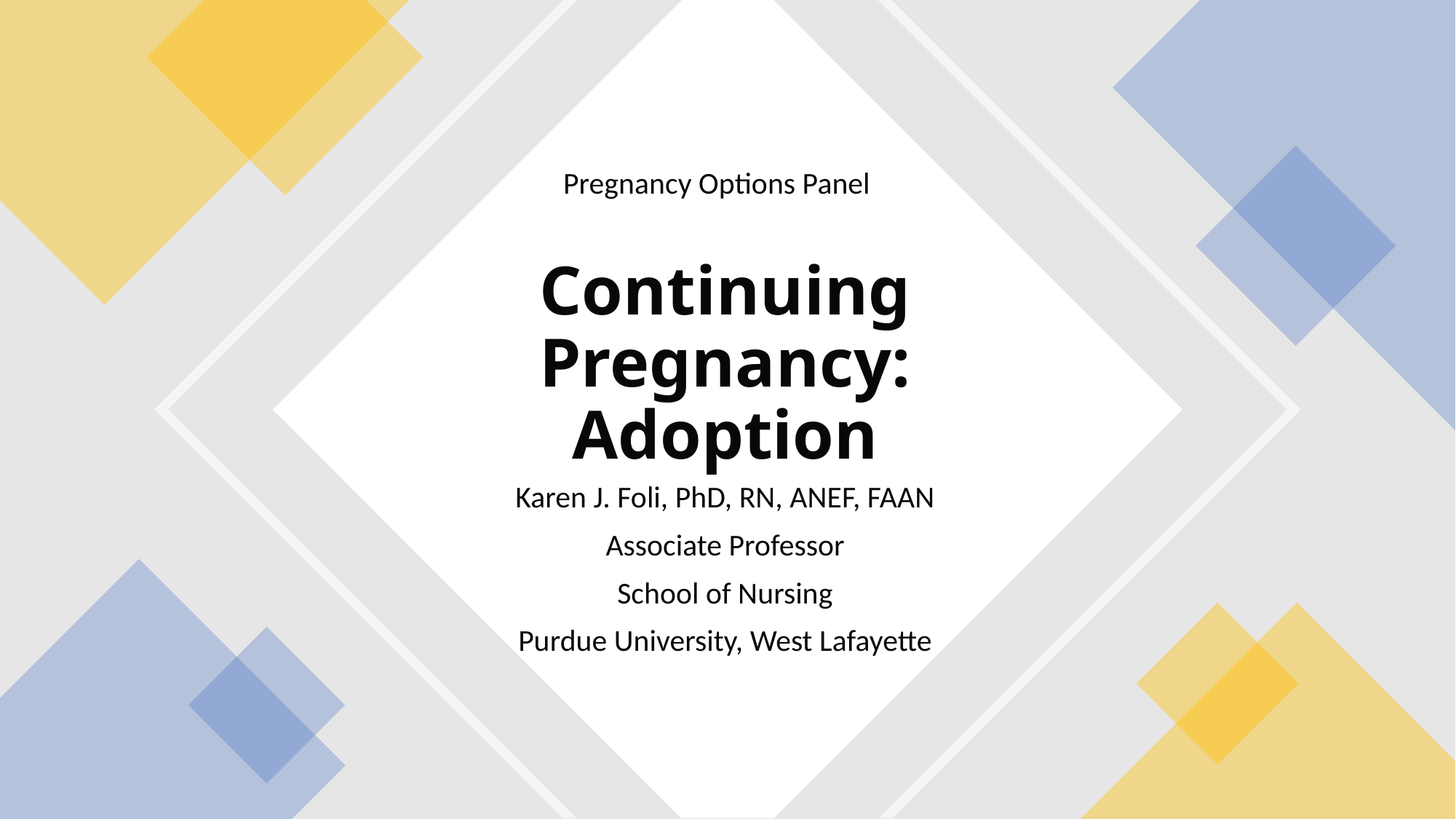

Pregnancy Options Panel
# Continuing Pregnancy: Adoption
Karen J. Foli, PhD, RN, ANEF, FAAN
Associate Professor
School of Nursing
Purdue University, West Lafayette

## Slide 2
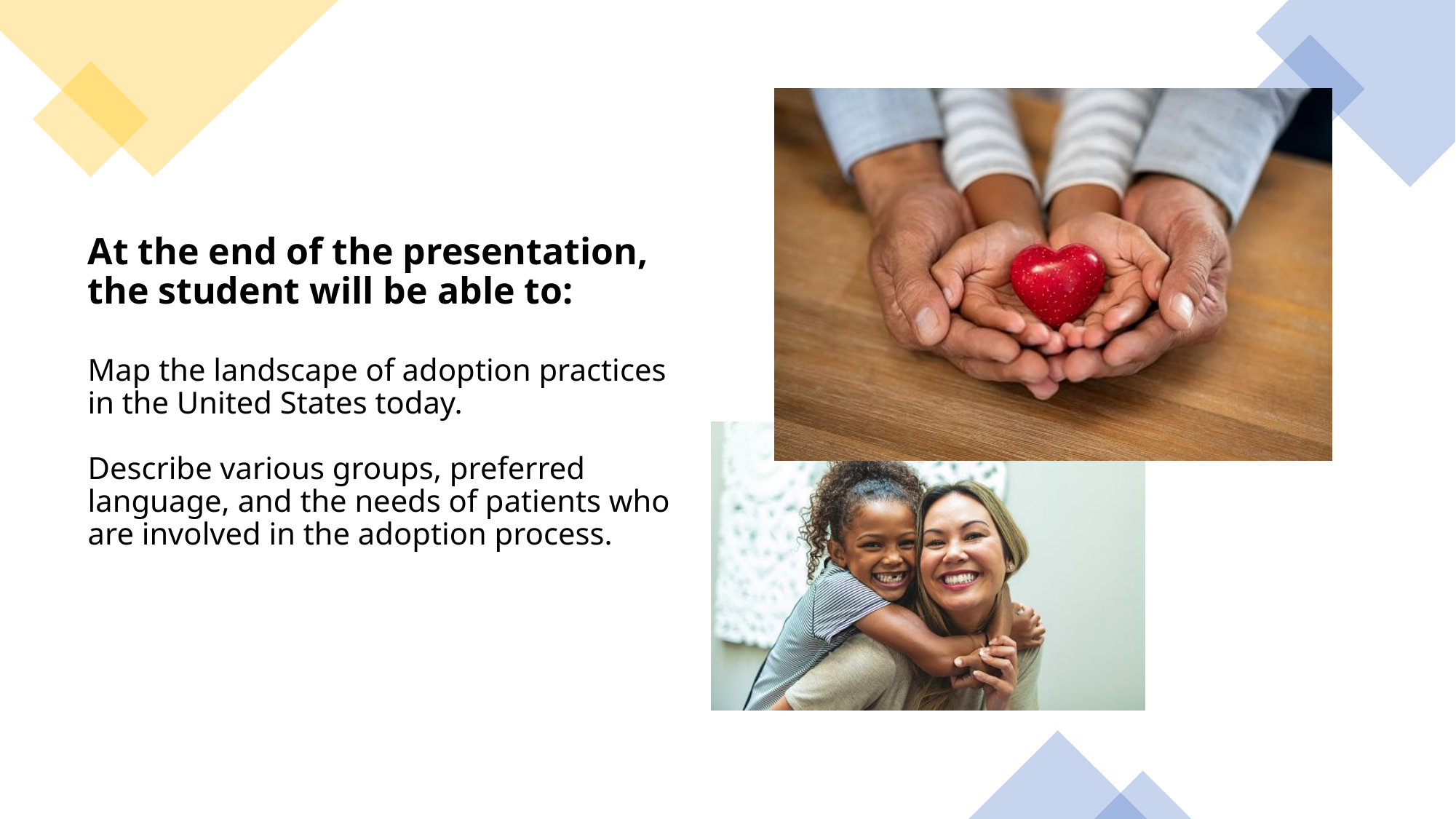

# At the end of the presentation, the student will be able to:
Map the landscape of adoption practices in the United States today.
Describe various groups, preferred language, and the needs of patients who are involved in the adoption process.

## Slide 3
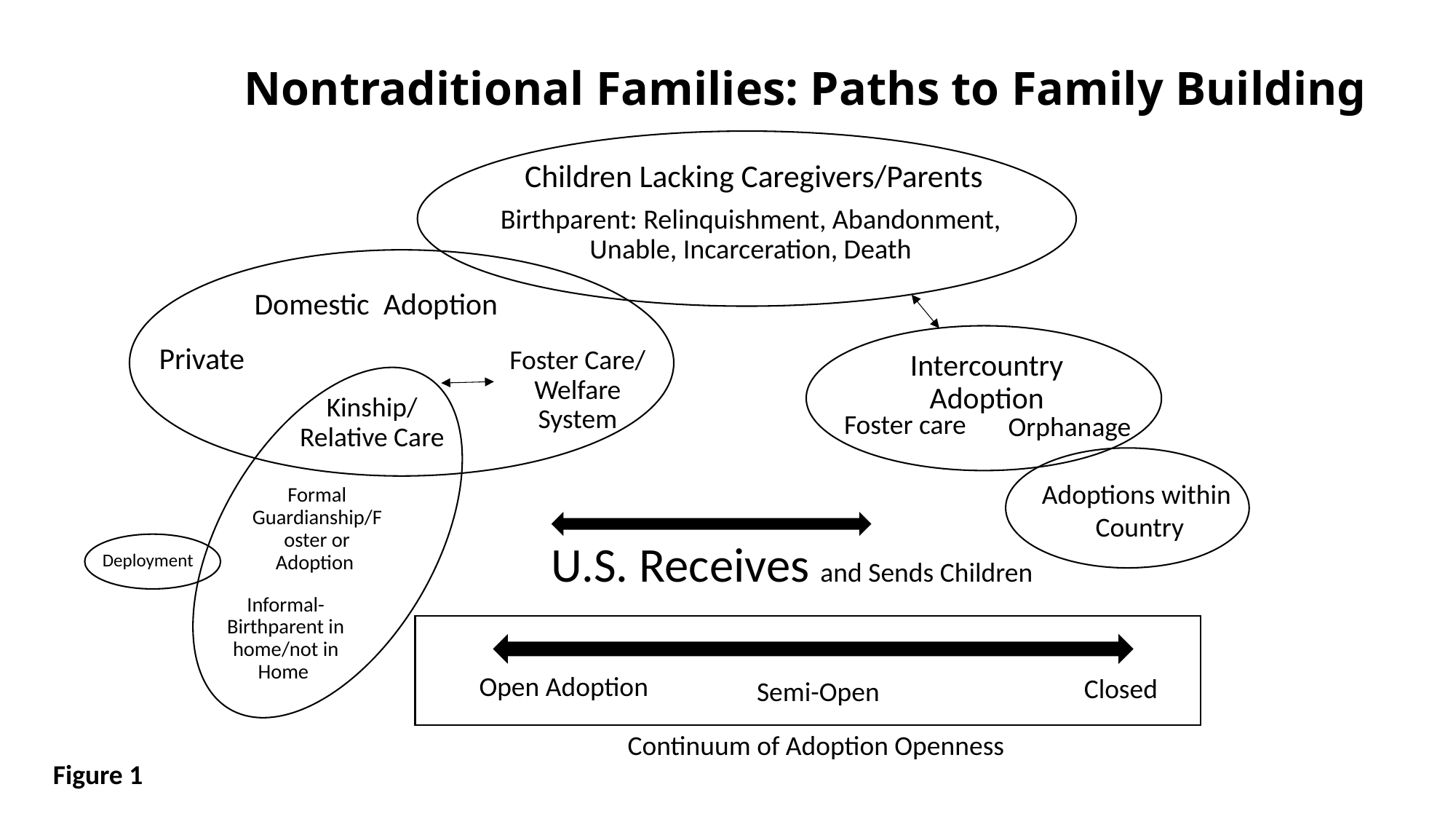

# Nontraditional Families: Paths to Family Building
 Children Lacking Caregivers/Parents
Birthparent: Relinquishment, Abandonment, Unable, Incarceration, Death
Private
Intercountry Adoption
Foster Care/
Welfare System
Kinship/
Relative Care
Orphanage
Foster care
Domestic Adoption
Adoptions within
Country
Formal Guardianship/Foster or Adoption
U.S. Receives and Sends Children
Deployment
Informal- Birthparent in home/not in Home
Open Adoption
Closed
Semi-Open
Continuum of Adoption Openness
Figure 1

## Slide 4
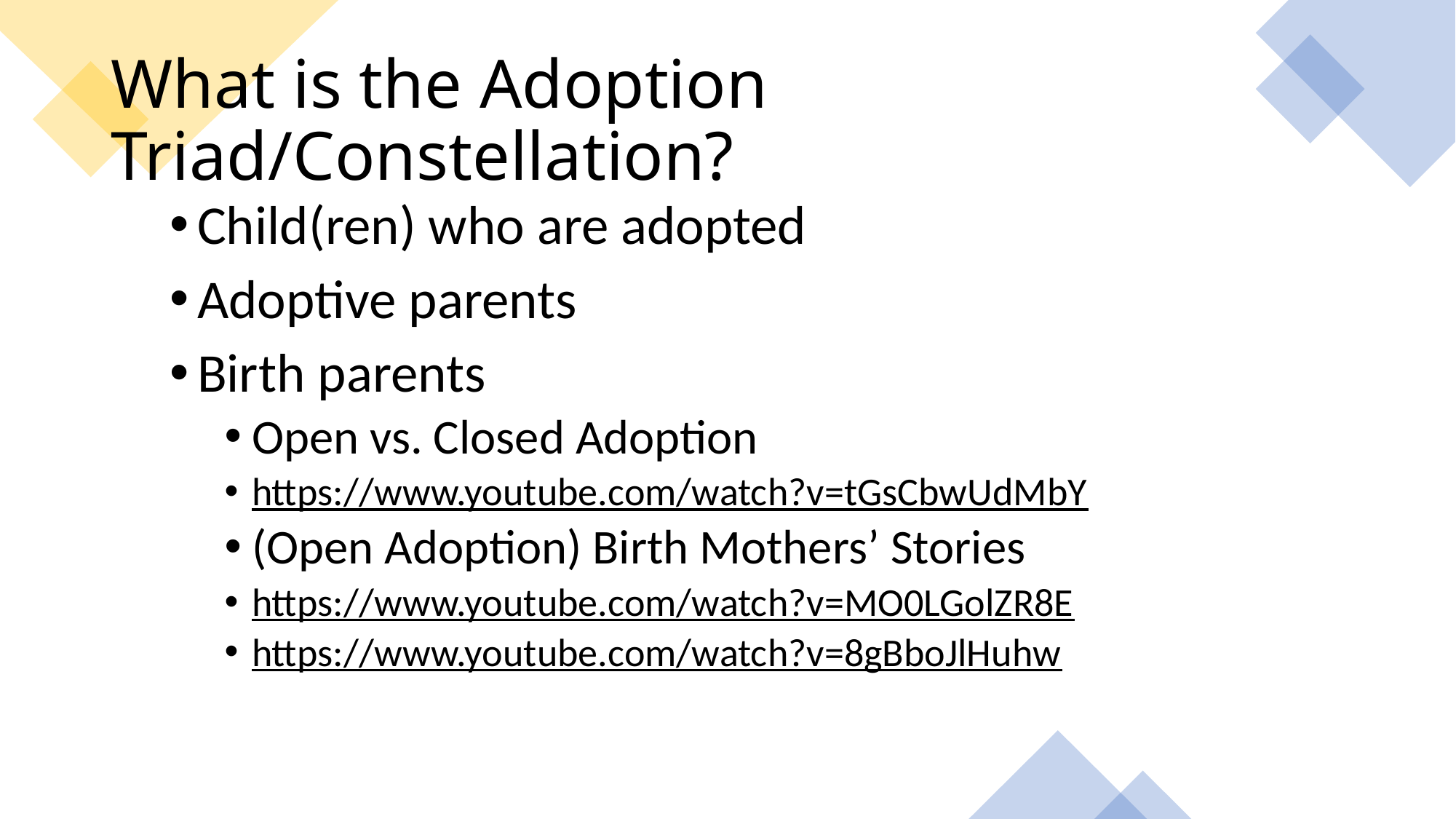

# What is the Adoption Triad/Constellation?
Child(ren) who are adopted
Adoptive parents
Birth parents
Open vs. Closed Adoption
https://www.youtube.com/watch?v=tGsCbwUdMbY
(Open Adoption) Birth Mothers’ Stories
https://www.youtube.com/watch?v=MO0LGolZR8E
https://www.youtube.com/watch?v=8gBboJlHuhw

## Slide 5
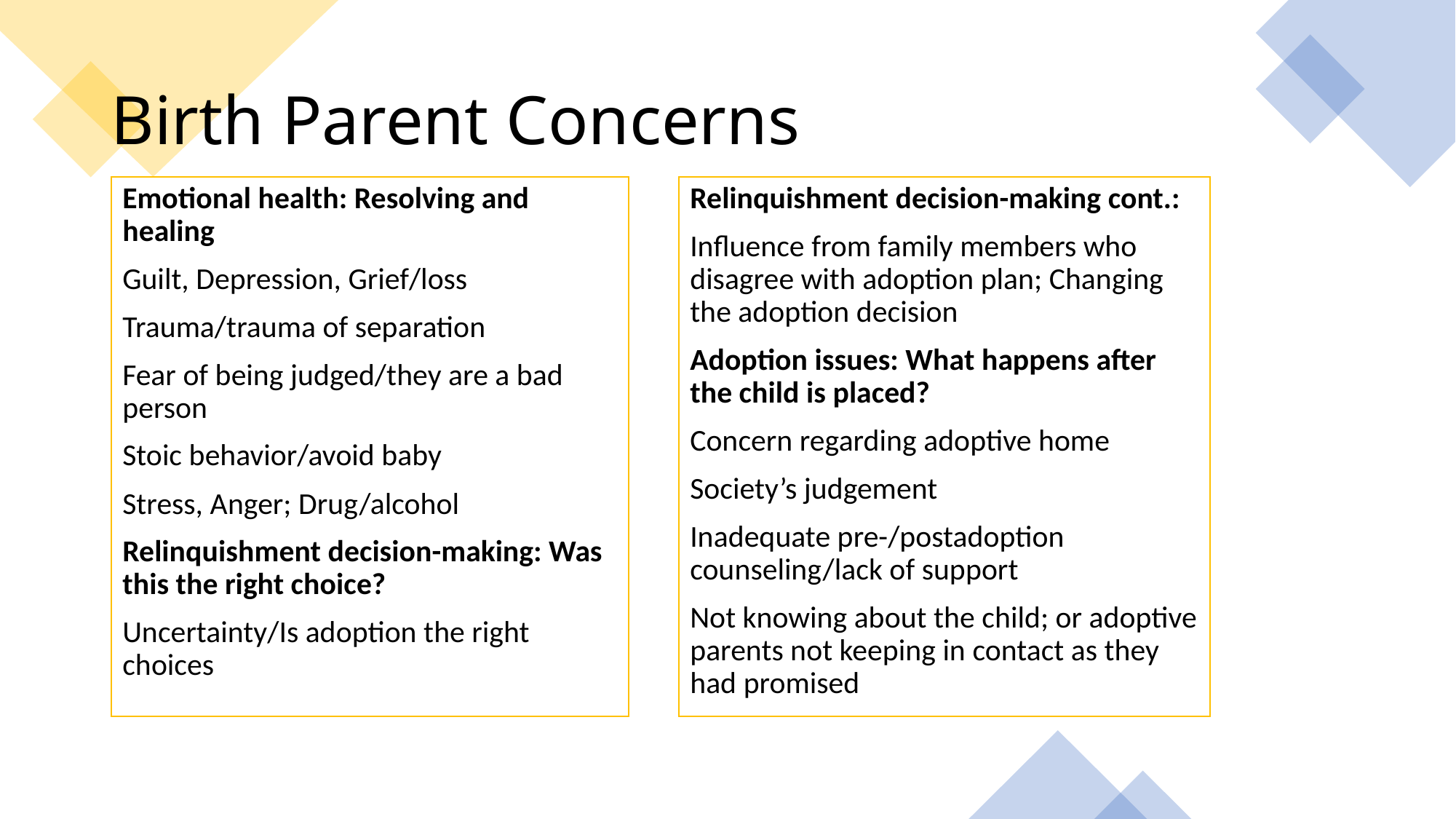

# Birth Parent Concerns
Emotional health: Resolving and healing
Guilt, Depression, Grief/loss
Trauma/trauma of separation
Fear of being judged/they are a bad person
Stoic behavior/avoid baby
Stress, Anger; Drug/alcohol
Relinquishment decision-making: Was this the right choice?
Uncertainty/Is adoption the right choices
Relinquishment decision-making cont.:
Influence from family members who disagree with adoption plan; Changing the adoption decision
Adoption issues: What happens after the child is placed?
Concern regarding adoptive home
Society’s judgement
Inadequate pre-/postadoption counseling/lack of support
Not knowing about the child; or adoptive parents not keeping in contact as they had promised

## Slide 6
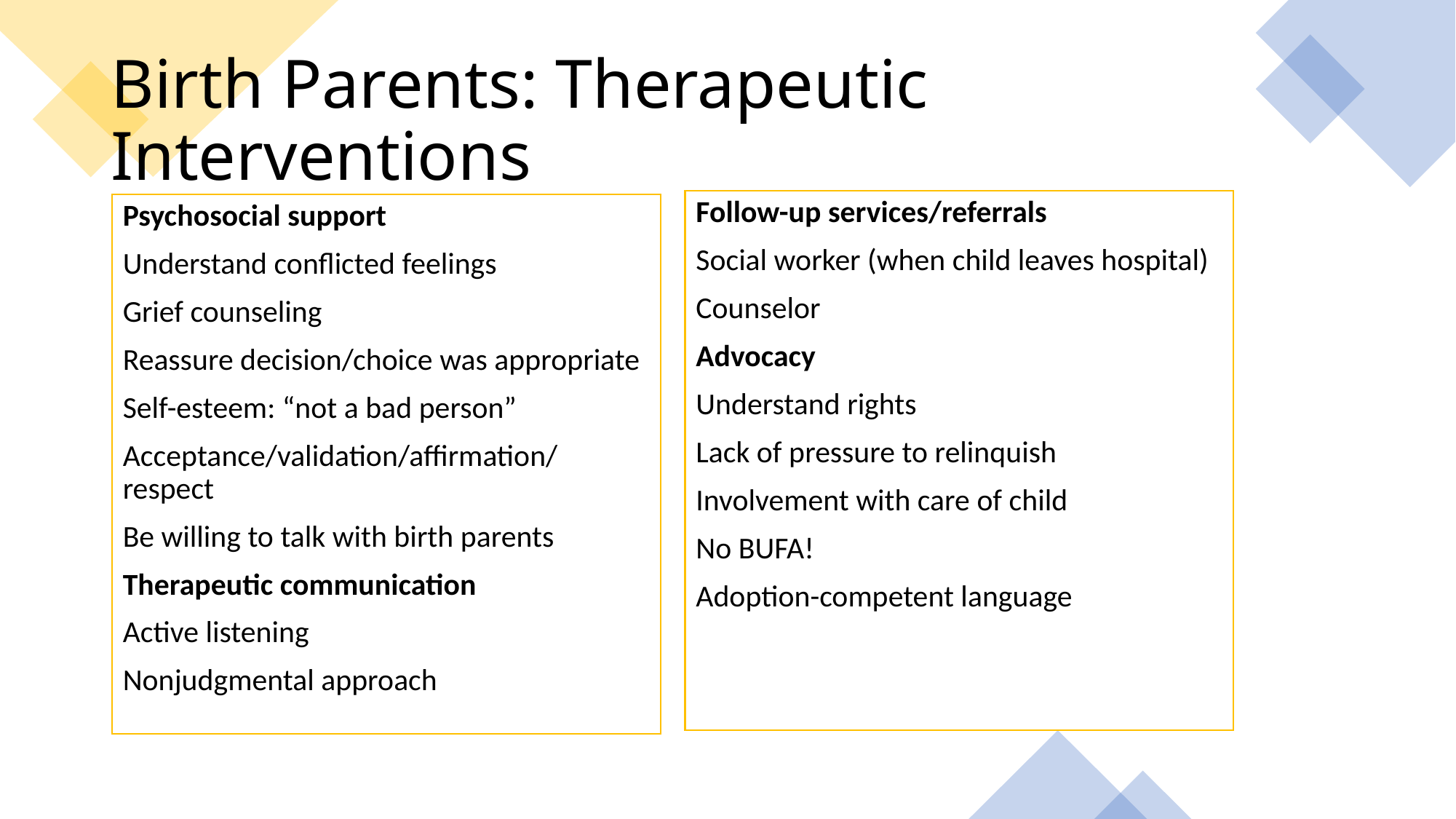

# Birth Parents: Therapeutic Interventions
Follow-up services/referrals
Social worker (when child leaves hospital)
Counselor
Advocacy
Understand rights
Lack of pressure to relinquish
Involvement with care of child
No BUFA!
Adoption-competent language
Psychosocial support
Understand conflicted feelings
Grief counseling
Reassure decision/choice was appropriate
Self-esteem: “not a bad person”
Acceptance/validation/affirmation/
respect
Be willing to talk with birth parents
Therapeutic communication
Active listening
Nonjudgmental approach

## Slide 7
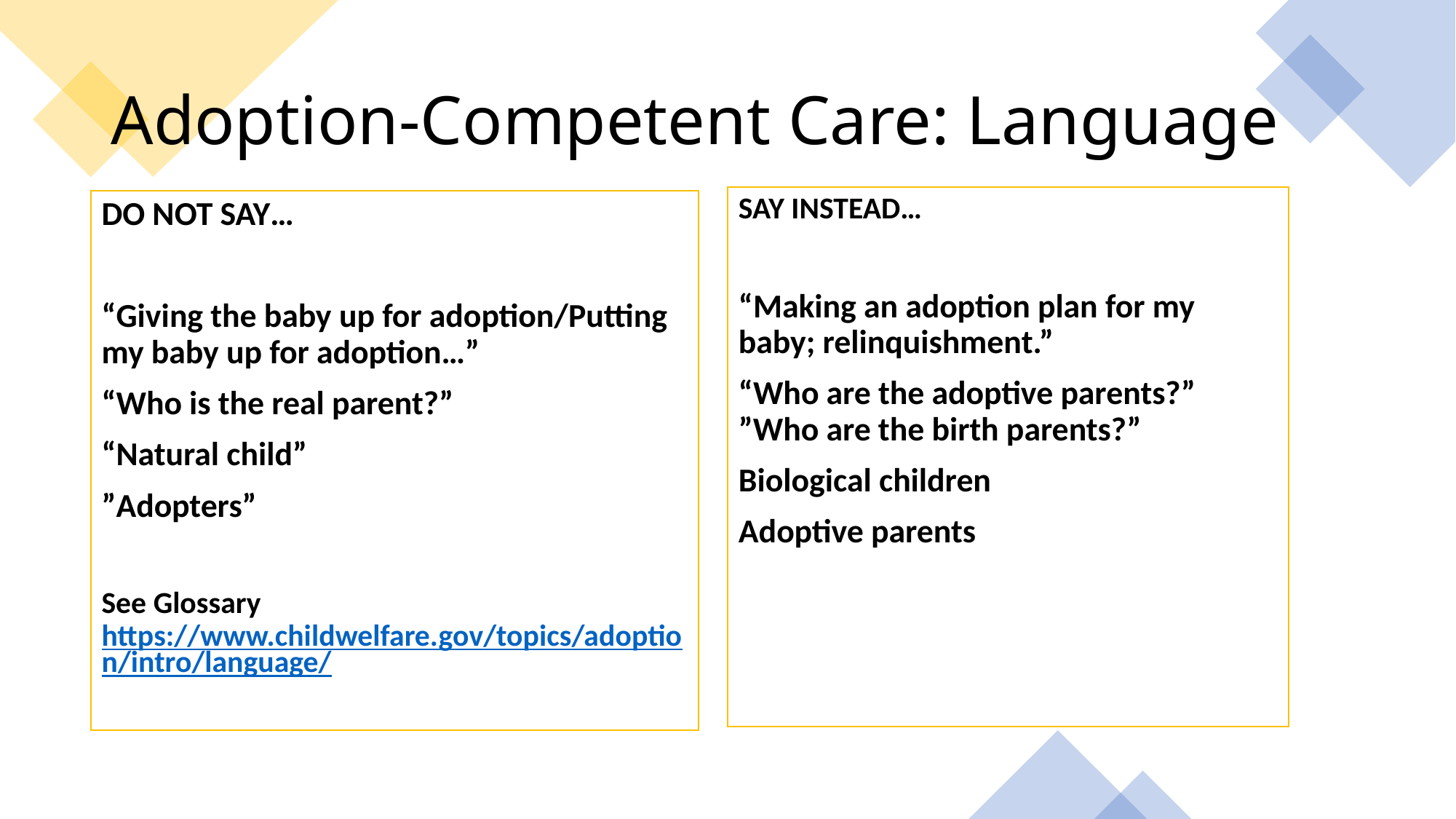

# Adoption-Competent Care: Language
SAY INSTEAD…
“Making an adoption plan for my baby; relinquishment.”
“Who are the adoptive parents?” ”Who are the birth parents?”
Biological children
Adoptive parents
DO NOT SAY…
“Giving the baby up for adoption/Putting my baby up for adoption…”
“Who is the real parent?”
“Natural child”
”Adopters”
See Glossary https://www.childwelfare.gov/topics/adoption/intro/language/

## Slide 8
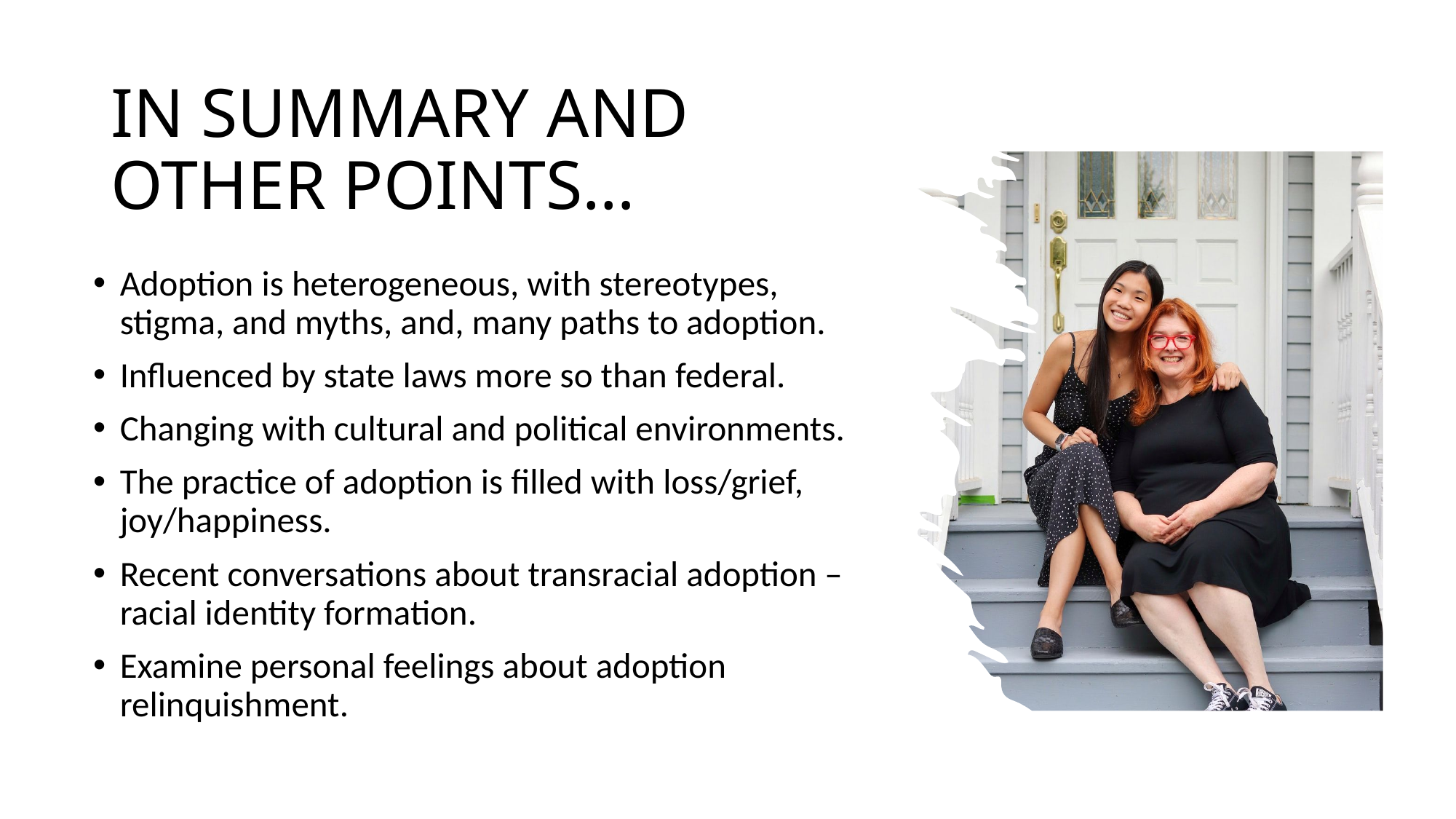

# IN SUMMARY AND OTHER POINTS…
Adoption is heterogeneous, with stereotypes, stigma, and myths, and, many paths to adoption.
Influenced by state laws more so than federal.
Changing with cultural and political environments.
The practice of adoption is filled with loss/grief, joy/happiness.
Recent conversations about transracial adoption – racial identity formation.
Examine personal feelings about adoption relinquishment.
