# Supplementary material for: Pregnancy options counseling in medical education and professionalism development
Source: AJOG Glob Rep. 2026 May 19;6(3):100656. doi: 10.1016/j.xagr.2026.100656 (PMC13314970; doi:10.1016/j.xagr.2026.100656)
Supplement: Supplementary file 3 [file mmc3.zip › mmc3.pptx]

## Slide 1
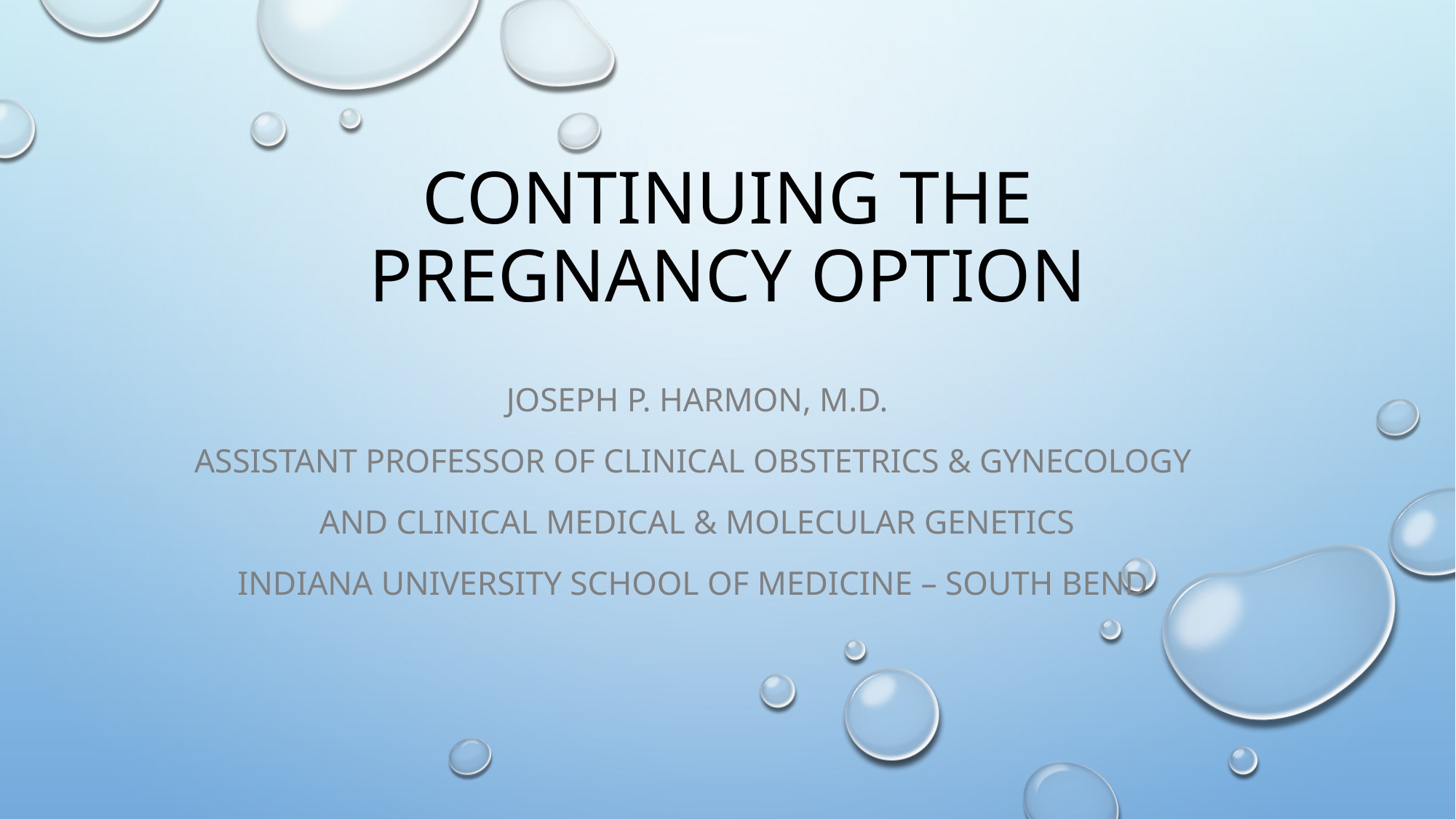

# CONTINUING THE PREGNANCY OPTION
 Joseph P. Harmon, M.D.
Assistant Professor of Clinical Obstetrics & Gynecology
 AND CLINICAL MEDICAL & MOLECULAR GENETICS
INDIANA UNIVERSITY SCHOOL OF MEDICINE – SOUTH BEND

## Slide 2
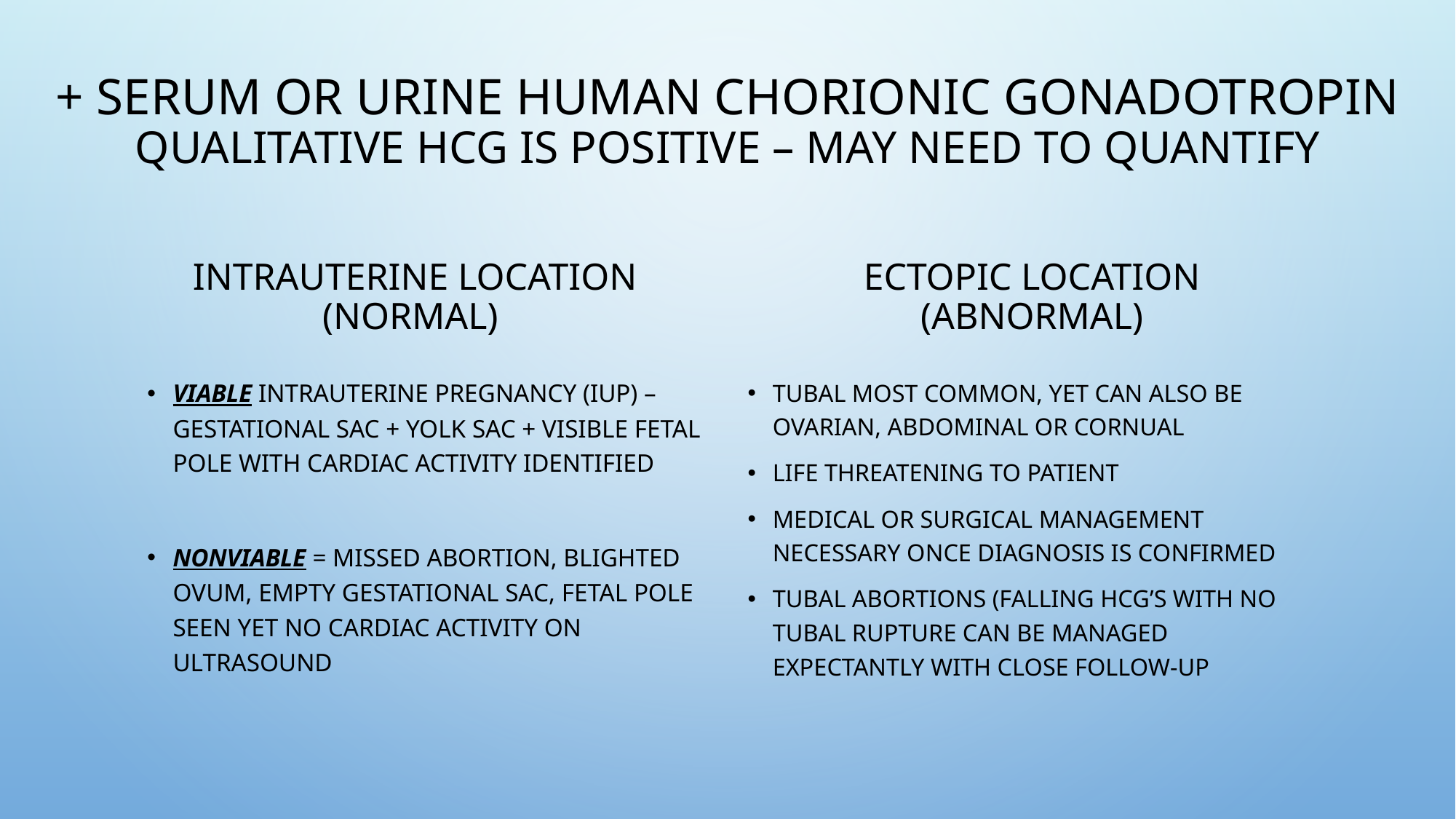

# + SERUM OR URINE HUMAN CHORIONIC GONADOTROPIN QUALITATIVE HCG is positive – may need to quantify
Ectopic location (abnormal)
Intrauterine location (normal)
Viable Intrauterine pregnancy (IUP) – gestational sac + yolk sac + visible fetal pole with cardiac activity identified
Nonviable = missed abortion, blighted ovum, empty gestational sac, fetal pole seen yet no cardiac activity on ultrasound
Tubal most common, yet can also be ovarian, abdominal or cornual
Life threatening to patient
Medical or surgical management necessary once diagnosis is confirmed
Tubal abortions (falling HCG’s with no tubal rupture can be managed expectantly with close follow-up

## Slide 3
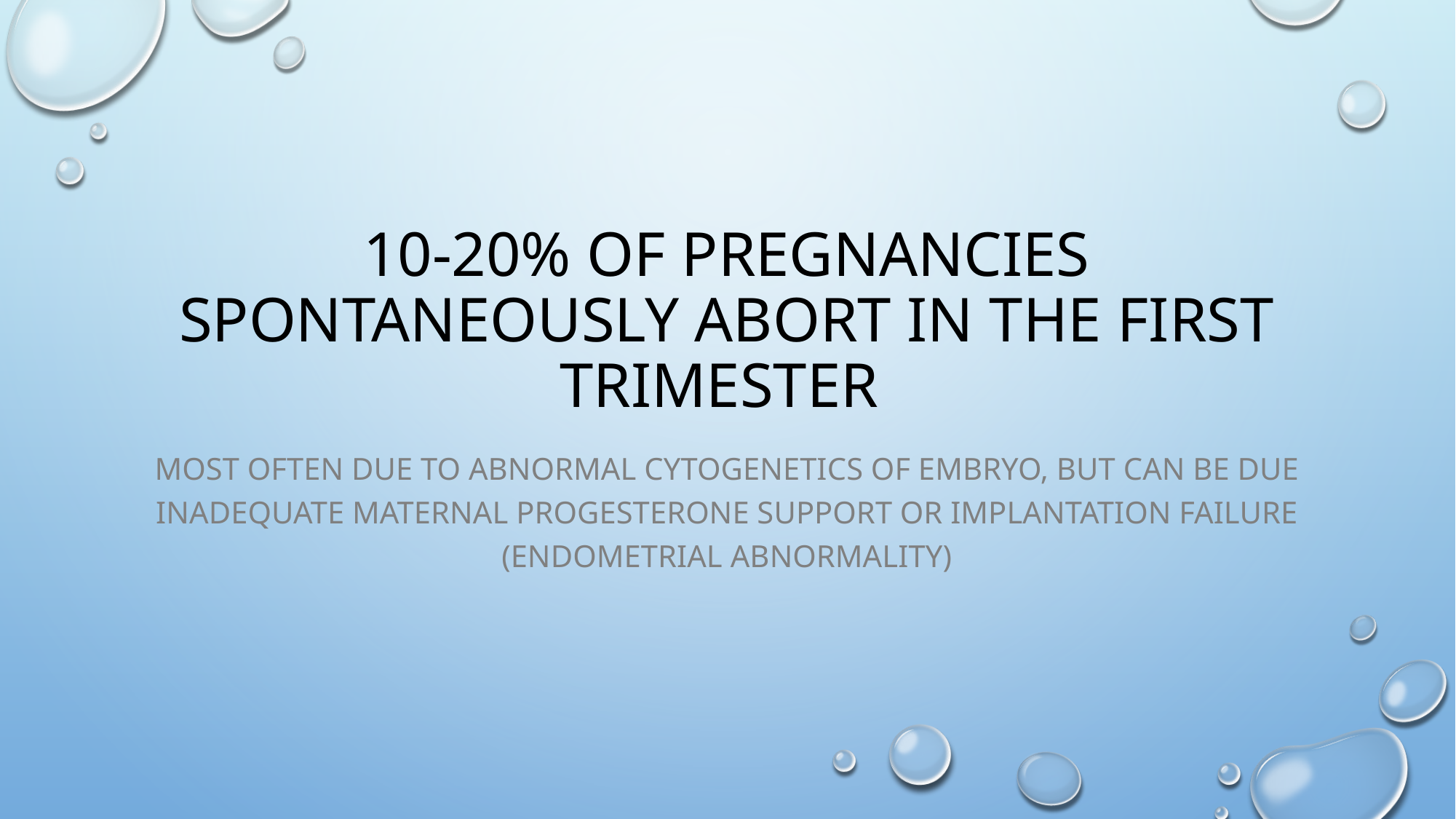

# 10-20% of pregnancies spontaneously abort in the first trimester
Most often due to abnormal cytogenetics of embryo, but can be due inadequate maternal progesterone support or implantation failure (endometrial abnormality)

## Slide 4
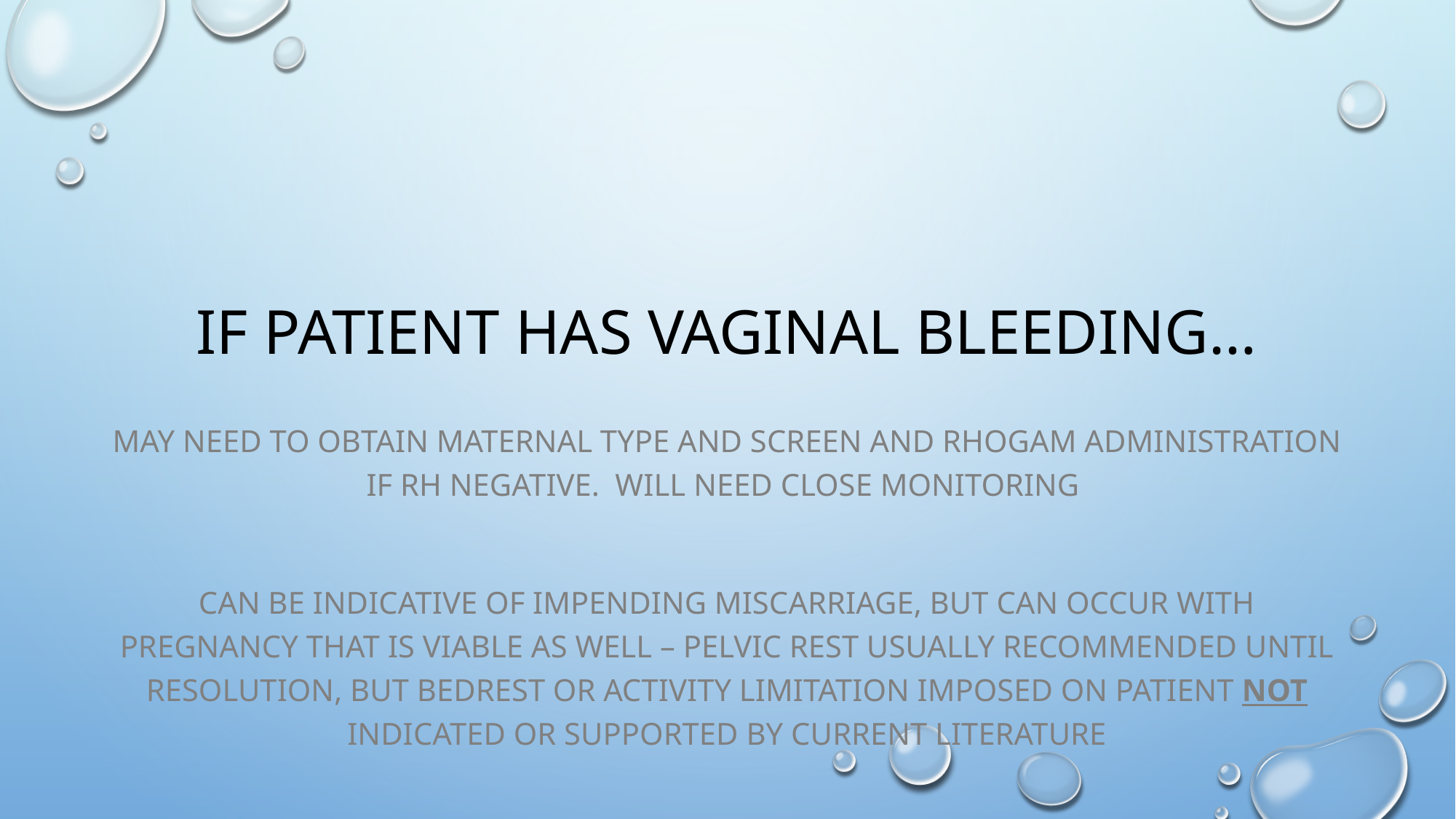

# If patient has vaginal bleeding…
May need to obtain maternal Type and Screen and Rhogam administration if Rh negative. Will need close monitoring
Can be indicative of impending miscarriage, but can occur with pregnancy that is viable as well – pelvic rest usually recommended until resolution, but bedrest or activity limitation imposed on patient not indicated or supported by current literature

## Slide 5
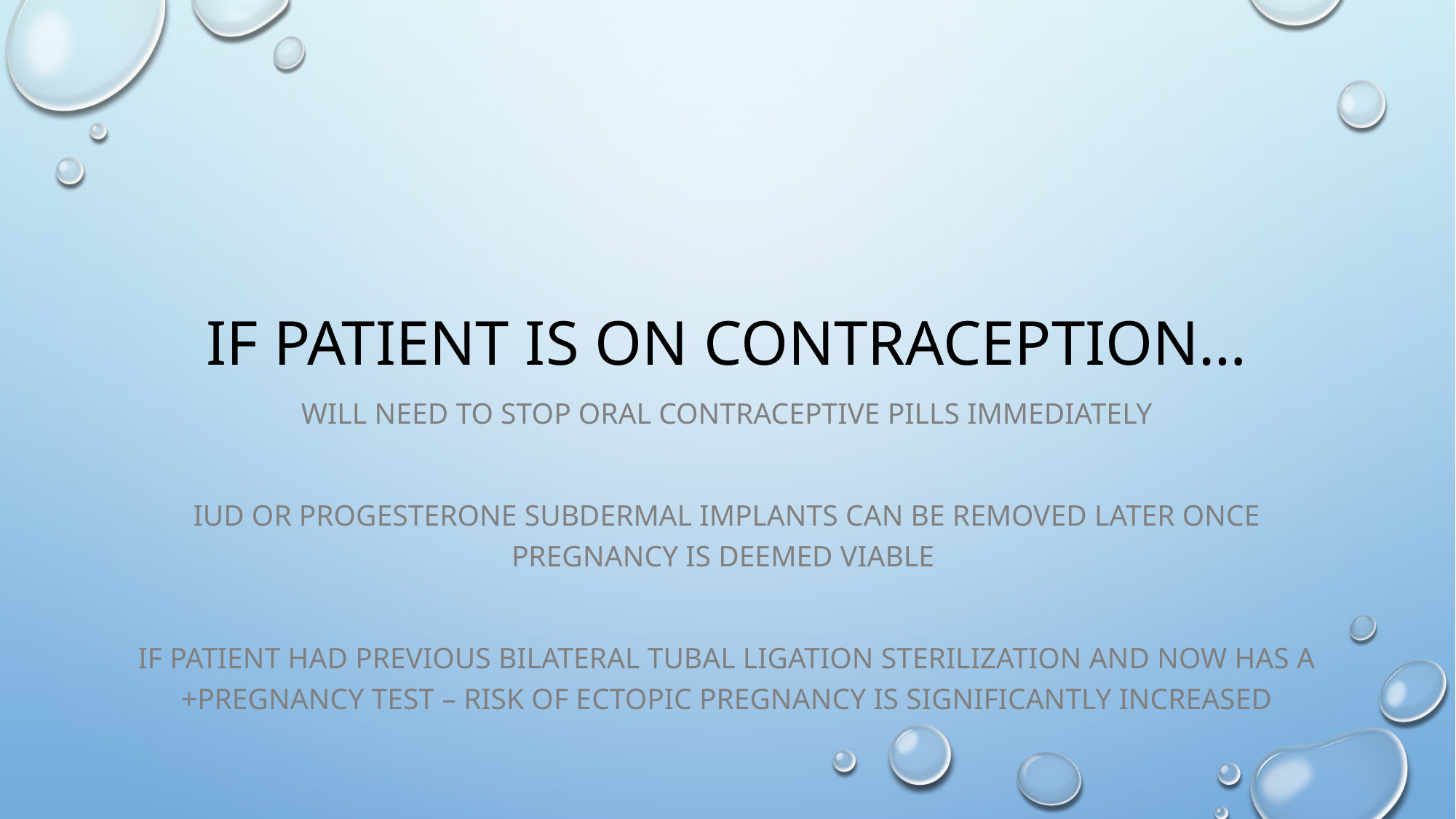

# If patient is on contraception…
Will need to stop oral contraceptive pills immediately
IUD or progesterone subdermal implants can be removed later once pregnancy is deemed viable
If patient had previous bilateral tubal ligation sterilization and now has a +pregnancy test – risk of ectopic pregnancy is significantly increased

## Slide 6
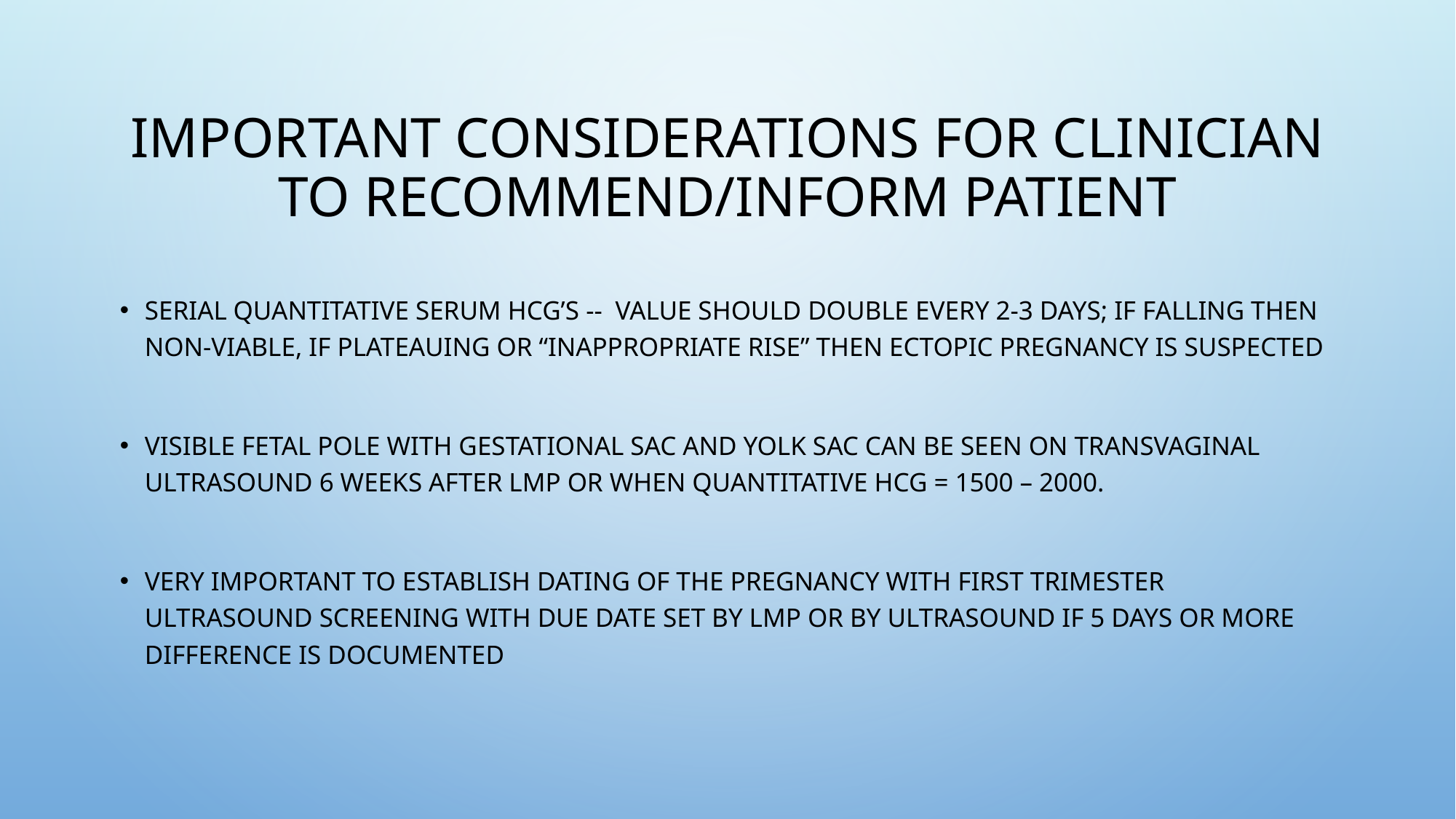

# Important considerations for clinician to recommend/inform patient
Serial quantitative serum HCG’s -- value should double every 2-3 days; if falling then non-viable, if plateauing or “inappropriate rise” then ectopic pregnancy is suspected
Visible fetal pole with gestational sac and yolk sac can be seen on transvaginal ultrasound 6 weeks after LMP or when quantitative HCG = 1500 – 2000.
Very important to establish dating of the pregnancy with first trimester ultrasound screening with due date set by LMP or by ultrasound if 5 days or more difference is documented

## Slide 7
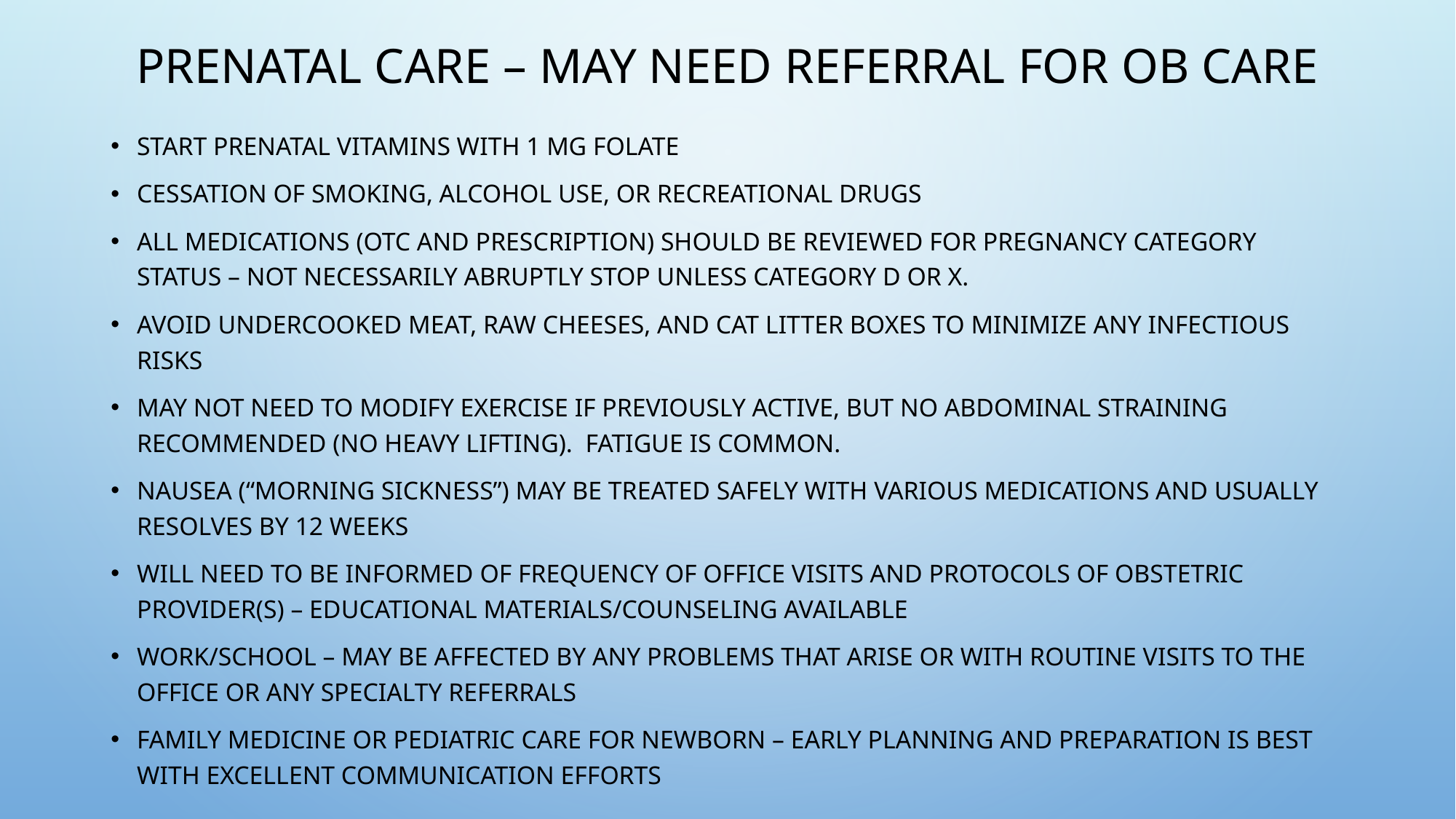

# Prenatal Care – may need referral for OB care
Start prenatal vitamins with 1 mg folate
Cessation of smoking, alcohol use, or recreational drugs
All medications (OTC and prescription) should be reviewed for Pregnancy category status – not necessarily abruptly stop unless Category D or X.
Avoid undercooked meat, raw cheeses, and cat litter boxes to minimize any infectious risks
May not need to modify exercise if previously active, but no abdominal straining recommended (no heavy lifting). Fatigue is common.
Nausea (“morning sickness”) may be treated safely with various medications and usually resolves by 12 weeks
Will need to be informed of frequency of office visits and protocols of obstetric provider(s) – educational materials/counseling available
Work/School – may be affected by any problems that arise or with routine visits to the office or any specialty referrals
Family medicine or pediatric care for newborn – early planning and preparation is best with excellent communication efforts

## Slide 8
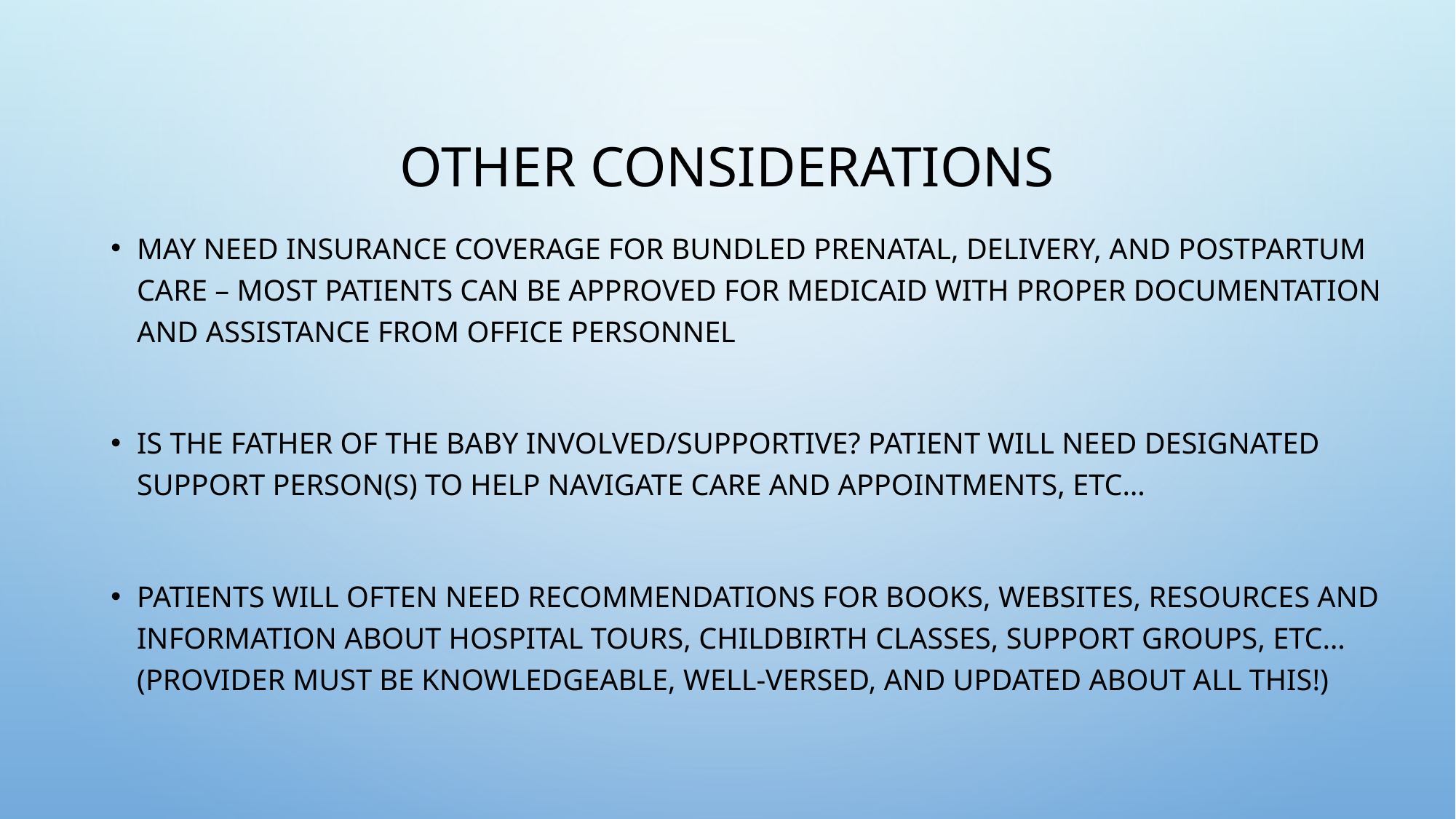

# Other considerations
May need insurance coverage for bundled prenatal, delivery, and postpartum care – most patients can be approved for Medicaid with proper documentation and assistance from office personnel
Is the father of the baby involved/supportive? Patient will need designated support person(s) to help navigate care and appointments, etc…
Patients will often need recommendations for books, websites, resources and information about hospital tours, childbirth classes, support groups, etc… (provider must be knowledgeable, well-versed, and updated about all this!)
