# Supplementary material for: Pregnancy options counseling in medical education and professionalism development
Source: AJOG Glob Rep. 2026 May 19;6(3):100656. doi: 10.1016/j.xagr.2026.100656 (PMC13314970; doi:10.1016/j.xagr.2026.100656)
Supplement: Supplementary file 4 [file mmc4.zip › mmc4.pptx]

## Slide 1
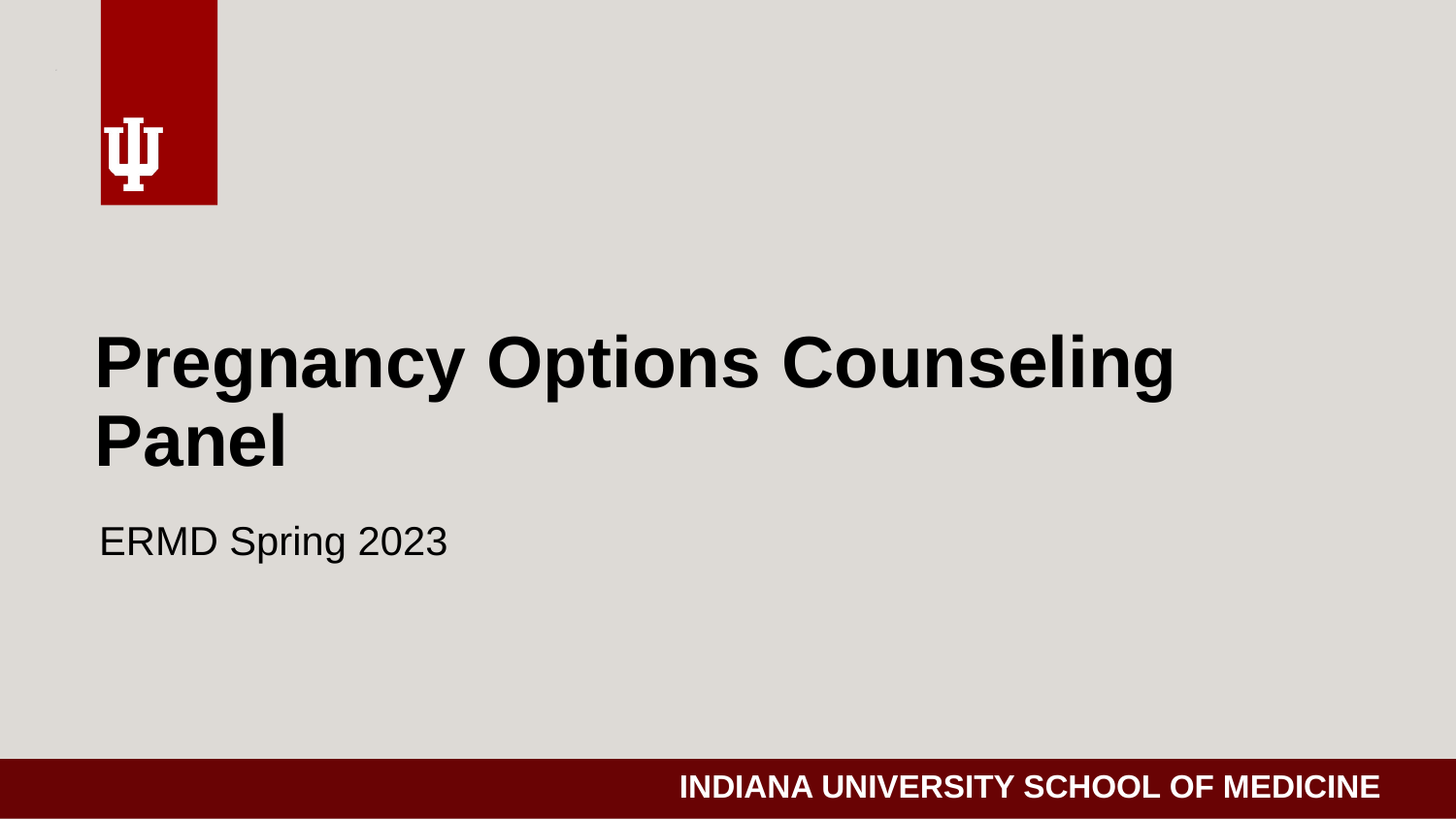

# Pregnancy Options Counseling Panel
ERMD Spring 2023

## Slide 2
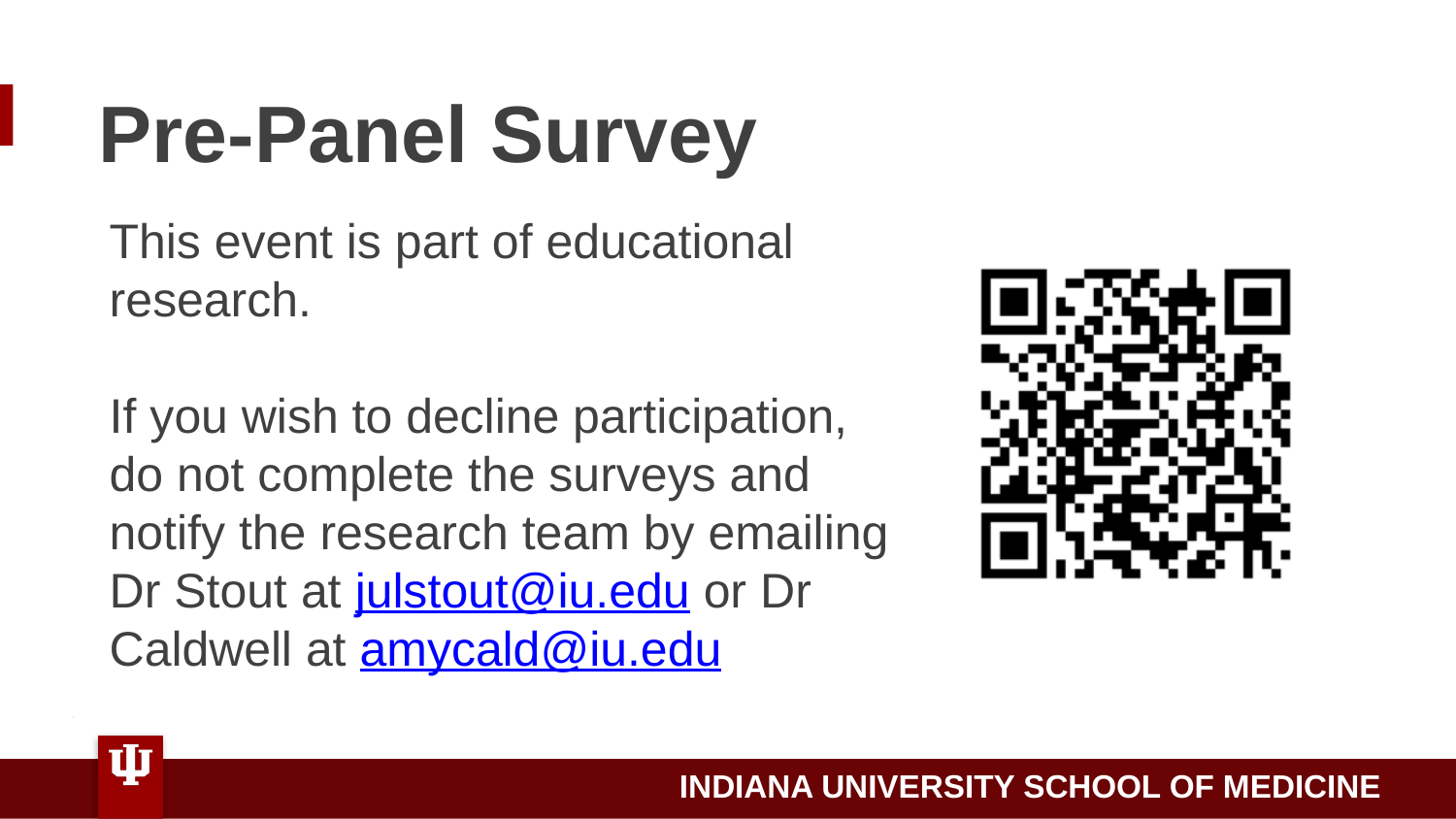

# Pre-Panel Survey
This event is part of educational research.
If you wish to decline participation, do not complete the surveys and notify the research team by emailing Dr Stout at julstout@iu.edu or Dr Caldwell at amycald@iu.edu

## Slide 3
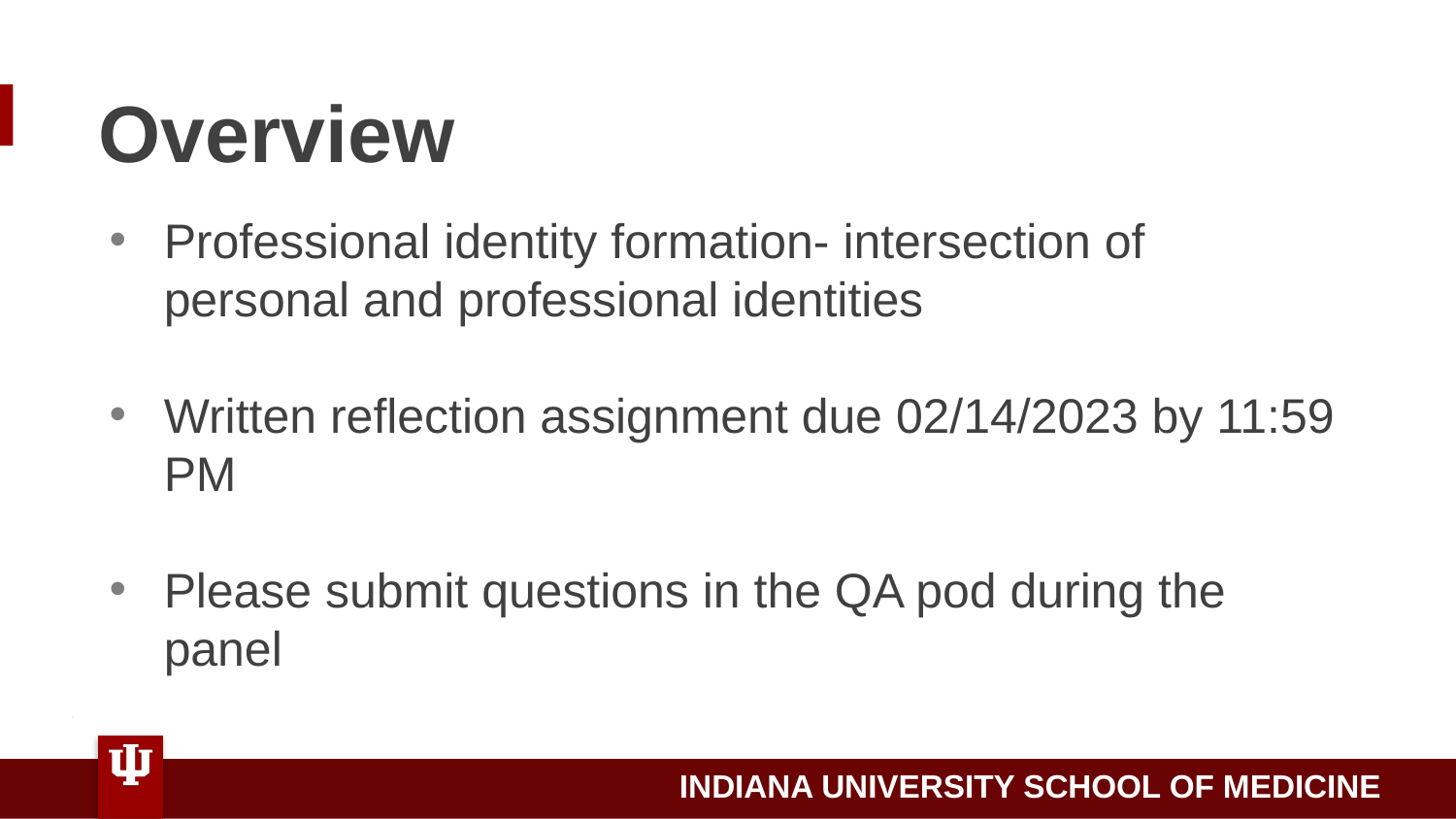

# Overview
Professional identity formation- intersection of personal and professional identities
Written reflection assignment due 02/14/2023 by 11:59 PM
Please submit questions in the QA pod during the panel

## Slide 4
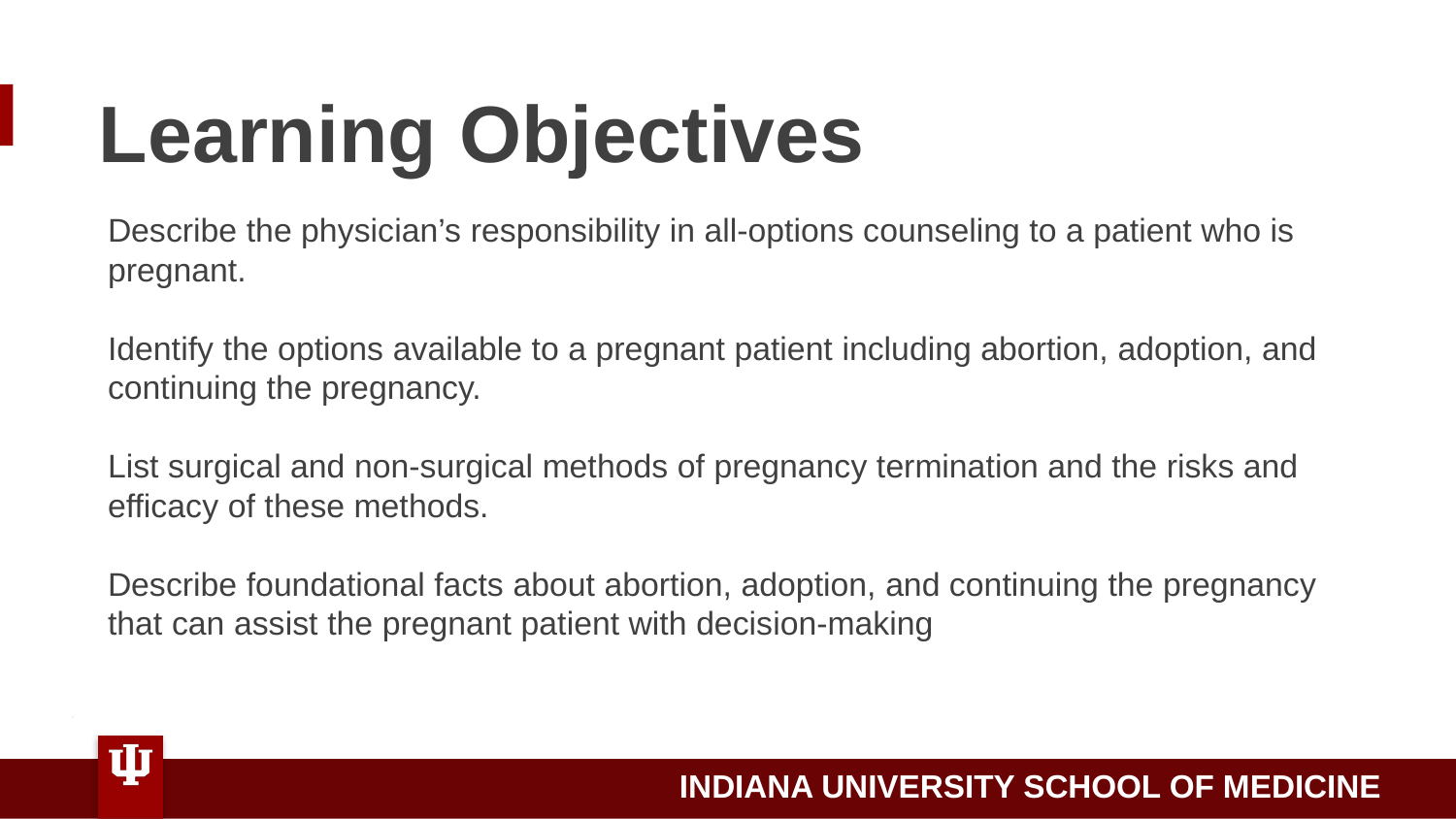

# Learning Objectives
Describe the physician’s responsibility in all-options counseling to a patient who is pregnant.
Identify the options available to a pregnant patient including abortion, adoption, and continuing the pregnancy.
List surgical and non-surgical methods of pregnancy termination and the risks and efficacy of these methods.
Describe foundational facts about abortion, adoption, and continuing the pregnancy that can assist the pregnant patient with decision-making

## Slide 5
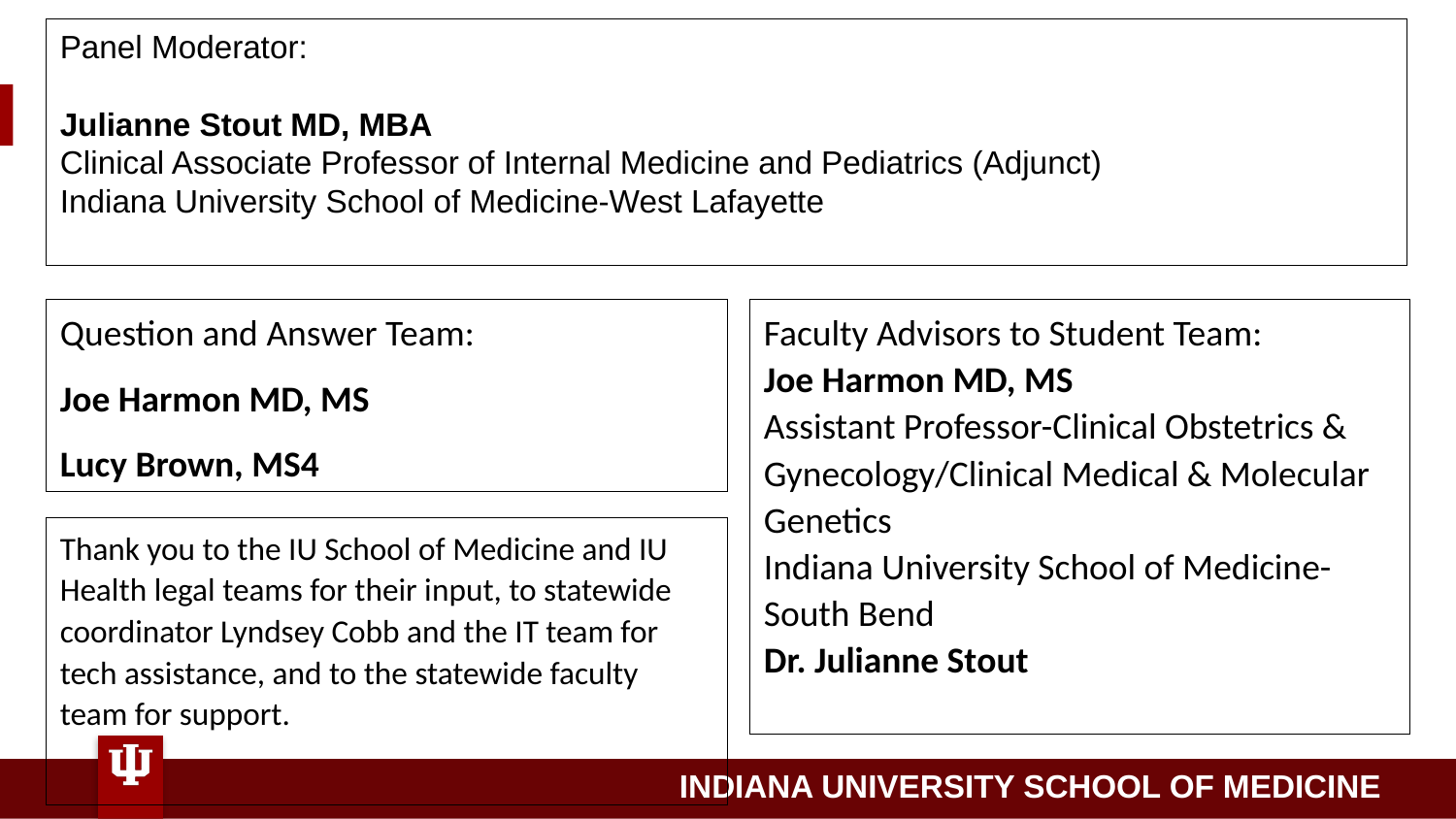

Panel Moderator:
Julianne Stout MD, MBA
Clinical Associate Professor of Internal Medicine and Pediatrics (Adjunct)
Indiana University School of Medicine-West Lafayette
Question and Answer Team:
Joe Harmon MD, MS
Lucy Brown, MS4
Faculty Advisors to Student Team:
Joe Harmon MD, MS
Assistant Professor-Clinical Obstetrics & Gynecology/Clinical Medical & Molecular Genetics
Indiana University School of Medicine-South Bend
Dr. Julianne Stout
Thank you to the IU School of Medicine and IU Health legal teams for their input, to statewide coordinator Lyndsey Cobb and the IT team for tech assistance, and to the statewide faculty team for support.

## Slide 6
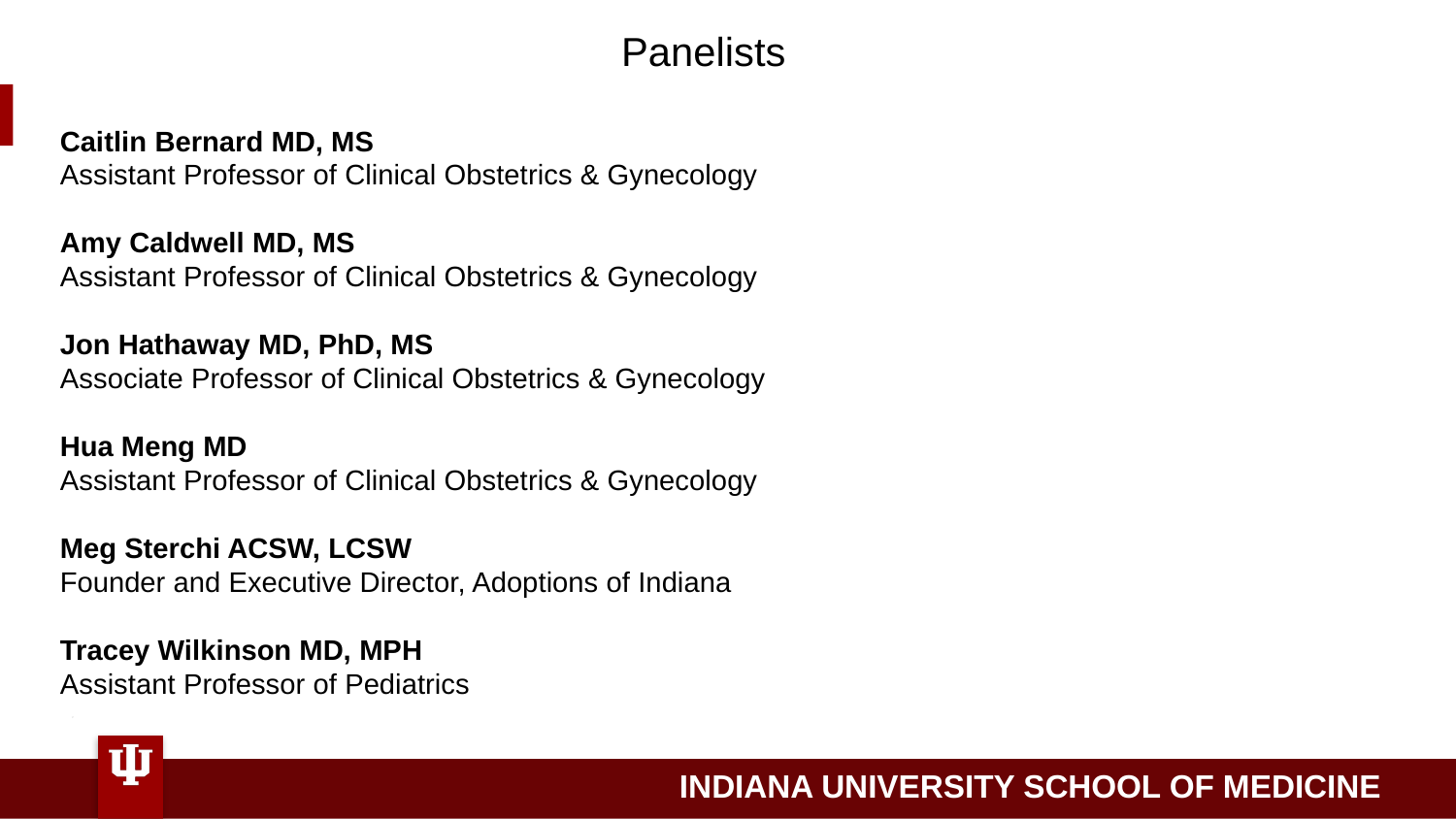

Panelists
Caitlin Bernard MD, MS
Assistant Professor of Clinical Obstetrics & Gynecology
Amy Caldwell MD, MS
Assistant Professor of Clinical Obstetrics & Gynecology
Jon Hathaway MD, PhD, MS
Associate Professor of Clinical Obstetrics & Gynecology
Hua Meng MD
Assistant Professor of Clinical Obstetrics & Gynecology
Meg Sterchi ACSW, LCSW
Founder and Executive Director, Adoptions of Indiana
Tracey Wilkinson MD, MPH
Assistant Professor of Pediatrics

## Slide 7
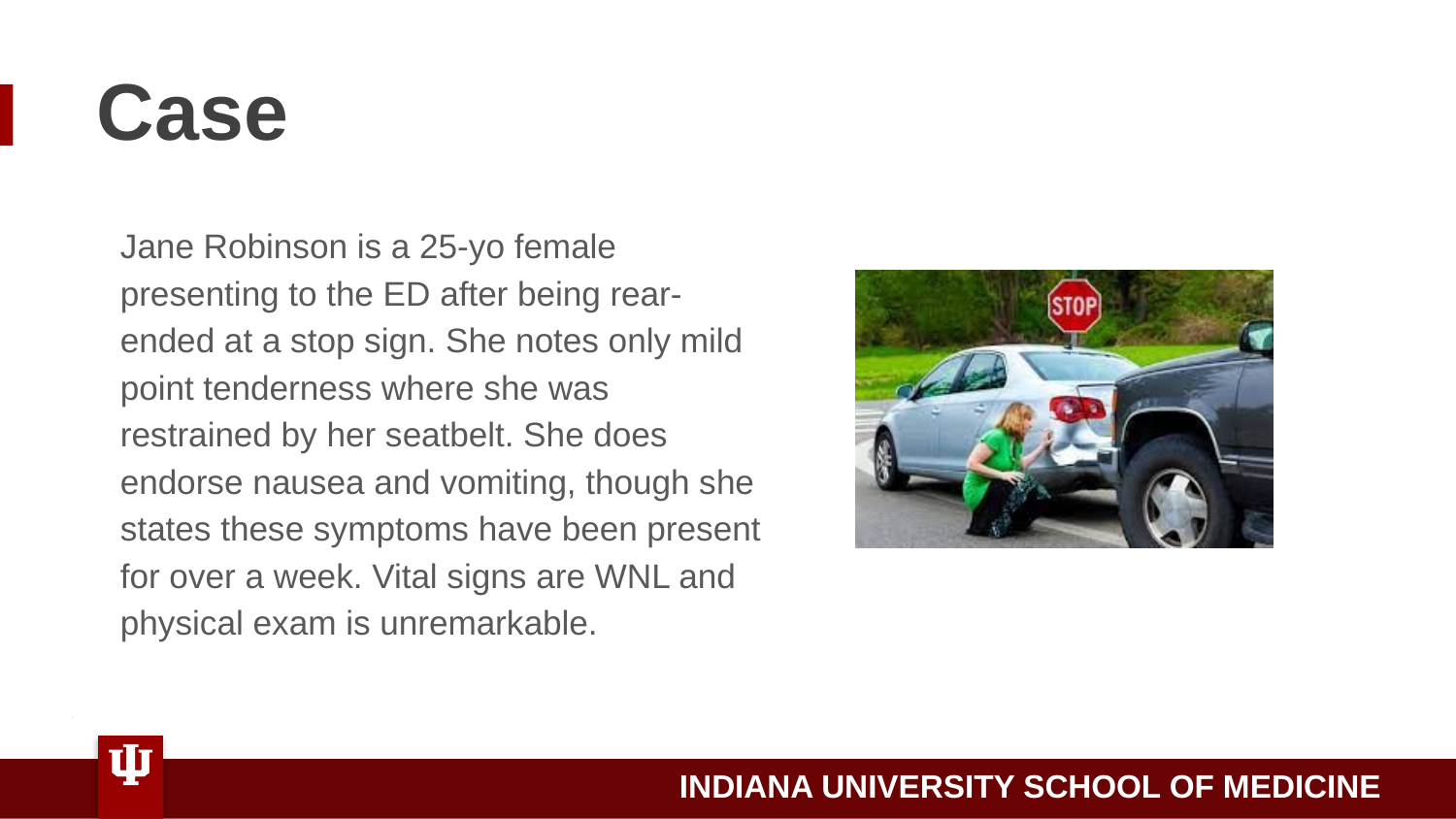

# Case
Jane Robinson is a 25-yo female presenting to the ED after being rear-ended at a stop sign. She notes only mild point tenderness where she was restrained by her seatbelt. She does endorse nausea and vomiting, though she states these symptoms have been present for over a week. Vital signs are WNL and physical exam is unremarkable.

## Slide 8
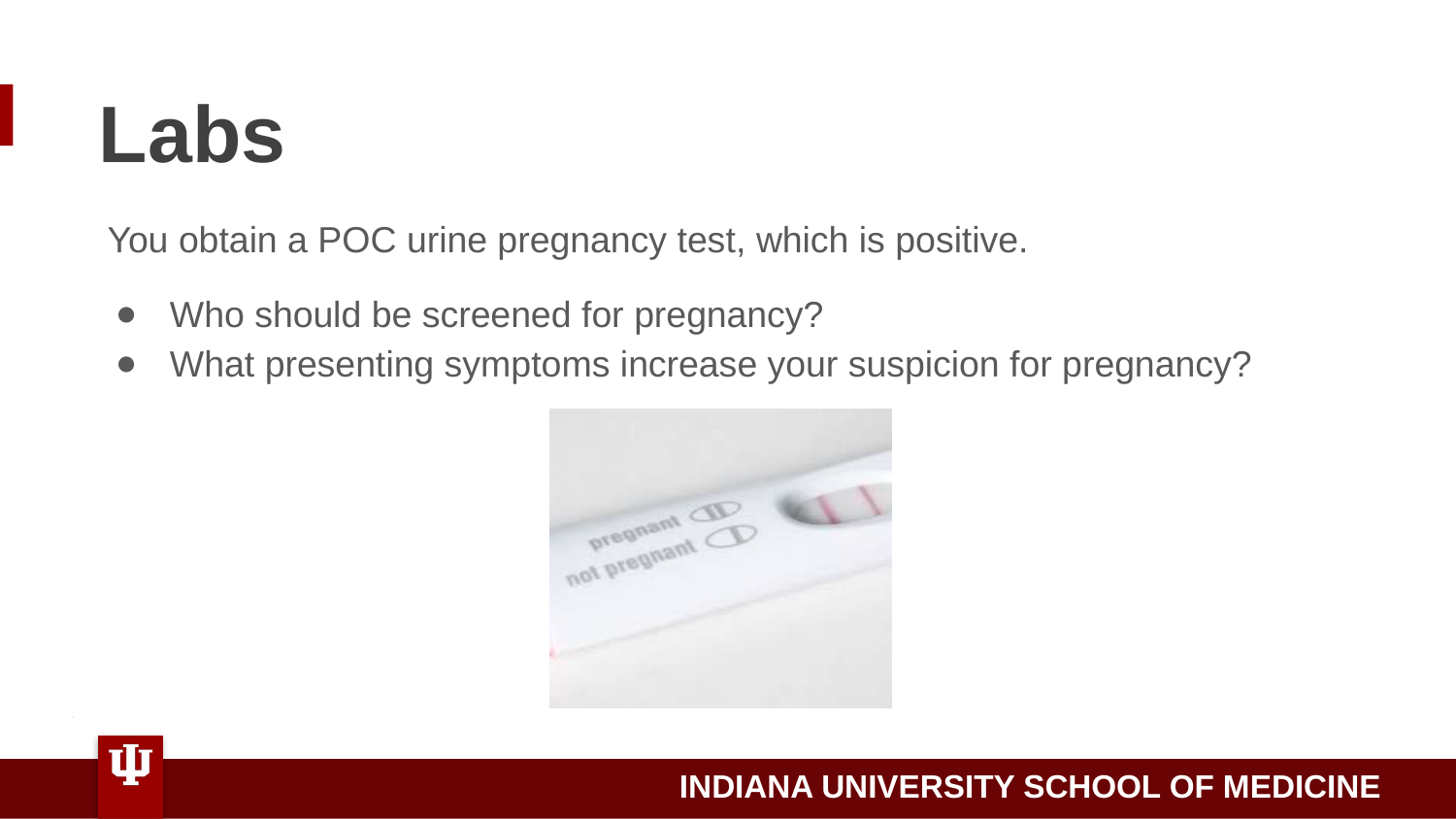

# Labs
 You obtain a POC urine pregnancy test, which is positive.
Who should be screened for pregnancy?
What presenting symptoms increase your suspicion for pregnancy?

## Slide 9
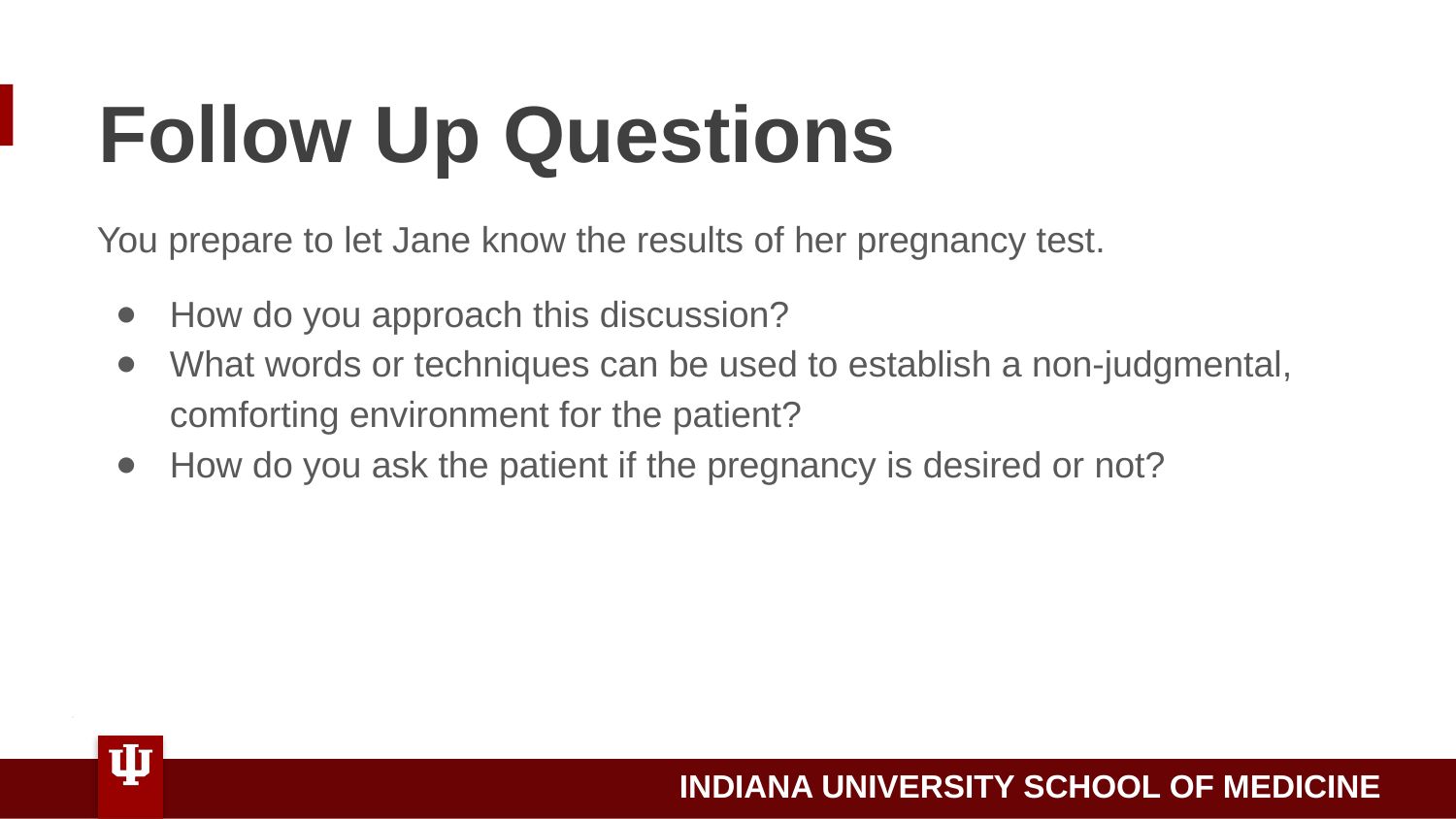

# Follow Up Questions
You prepare to let Jane know the results of her pregnancy test.
How do you approach this discussion?
What words or techniques can be used to establish a non-judgmental, comforting environment for the patient?
How do you ask the patient if the pregnancy is desired or not?

## Slide 10
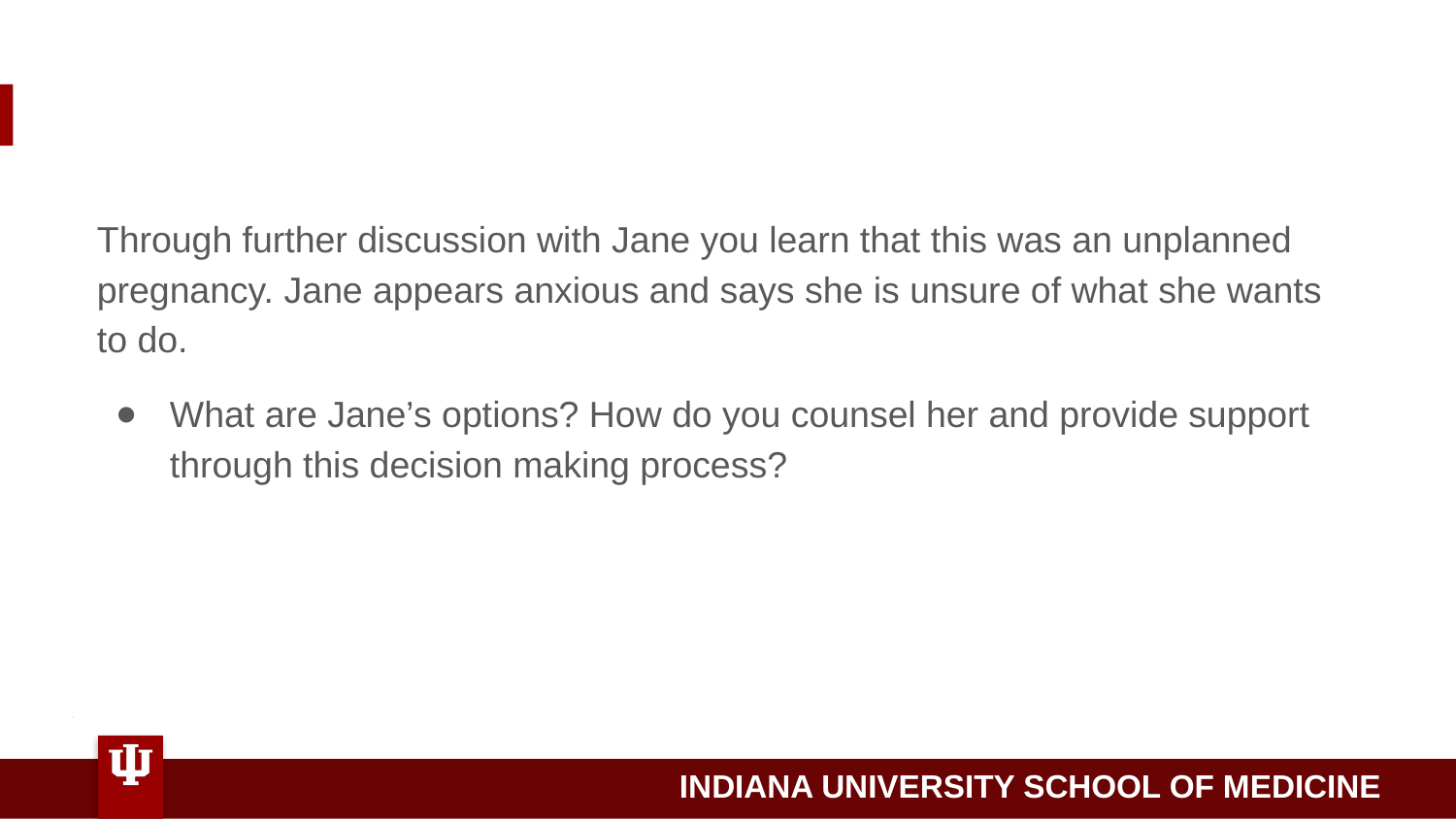

Through further discussion with Jane you learn that this was an unplanned pregnancy. Jane appears anxious and says she is unsure of what she wants to do.
What are Jane’s options? How do you counsel her and provide support through this decision making process?

## Slide 11
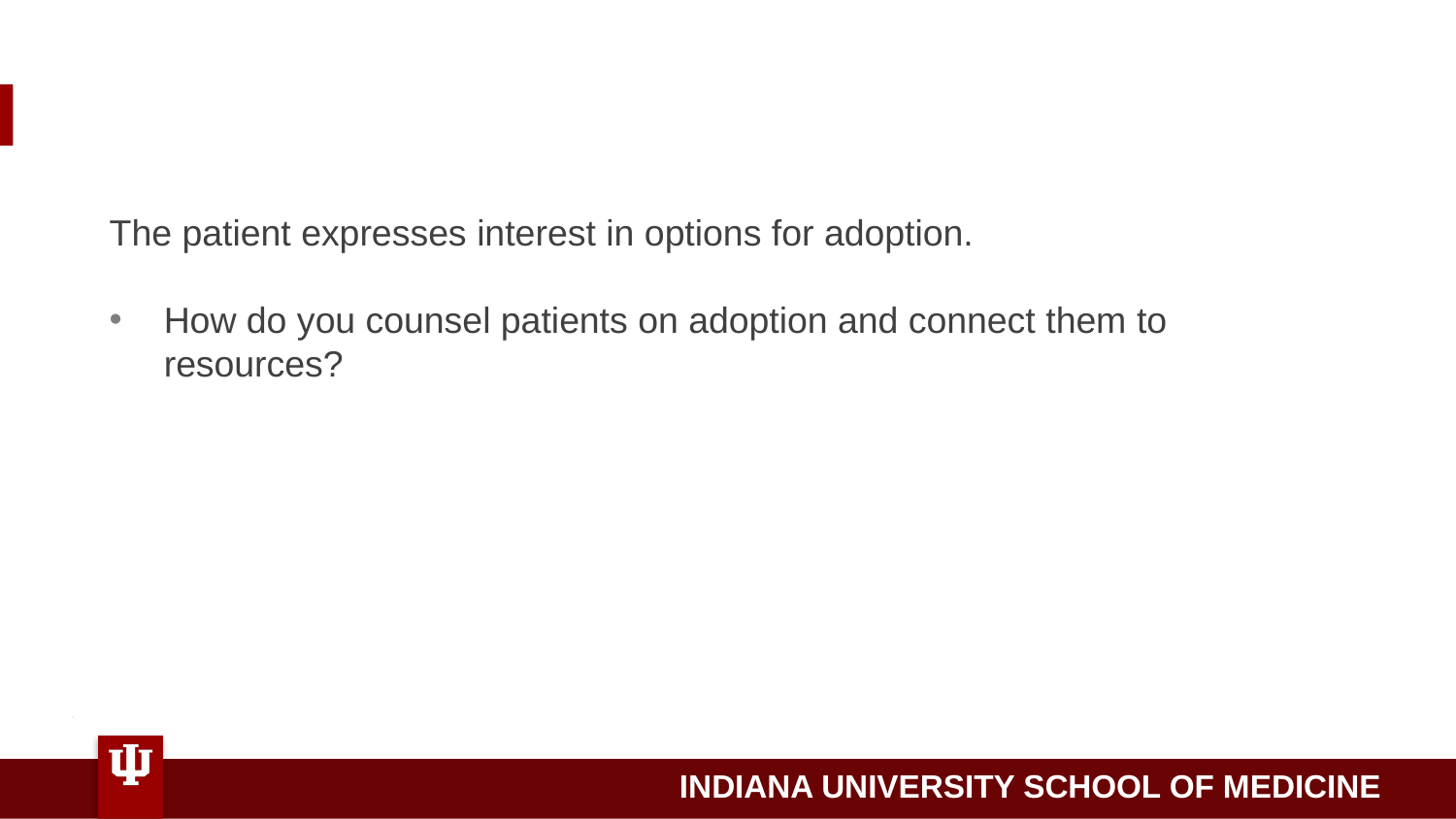

The patient expresses interest in options for adoption.
How do you counsel patients on adoption and connect them to resources?

## Slide 12
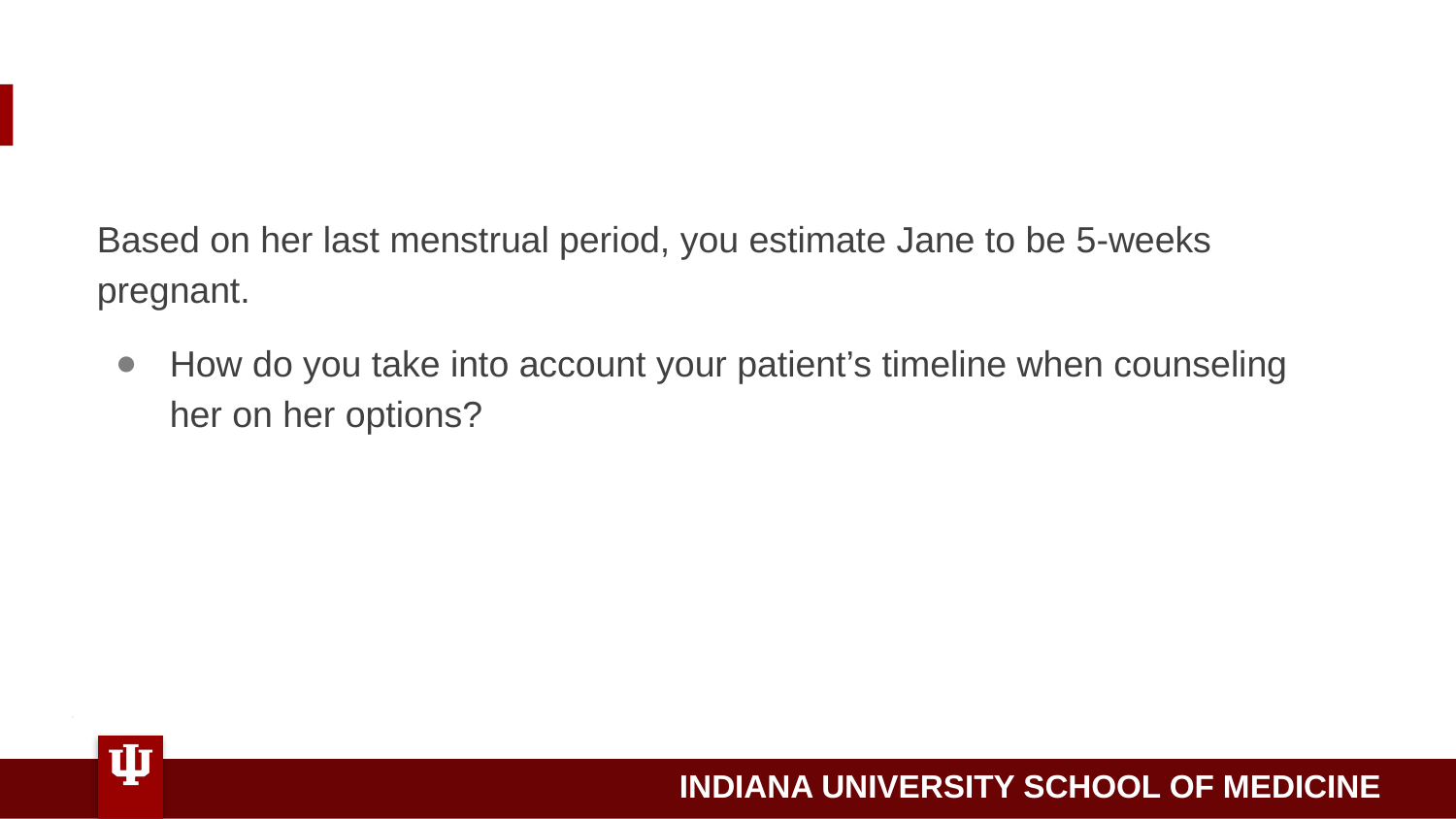

Based on her last menstrual period, you estimate Jane to be 5-weeks pregnant.
How do you take into account your patient’s timeline when counseling her on her options?

## Slide 13
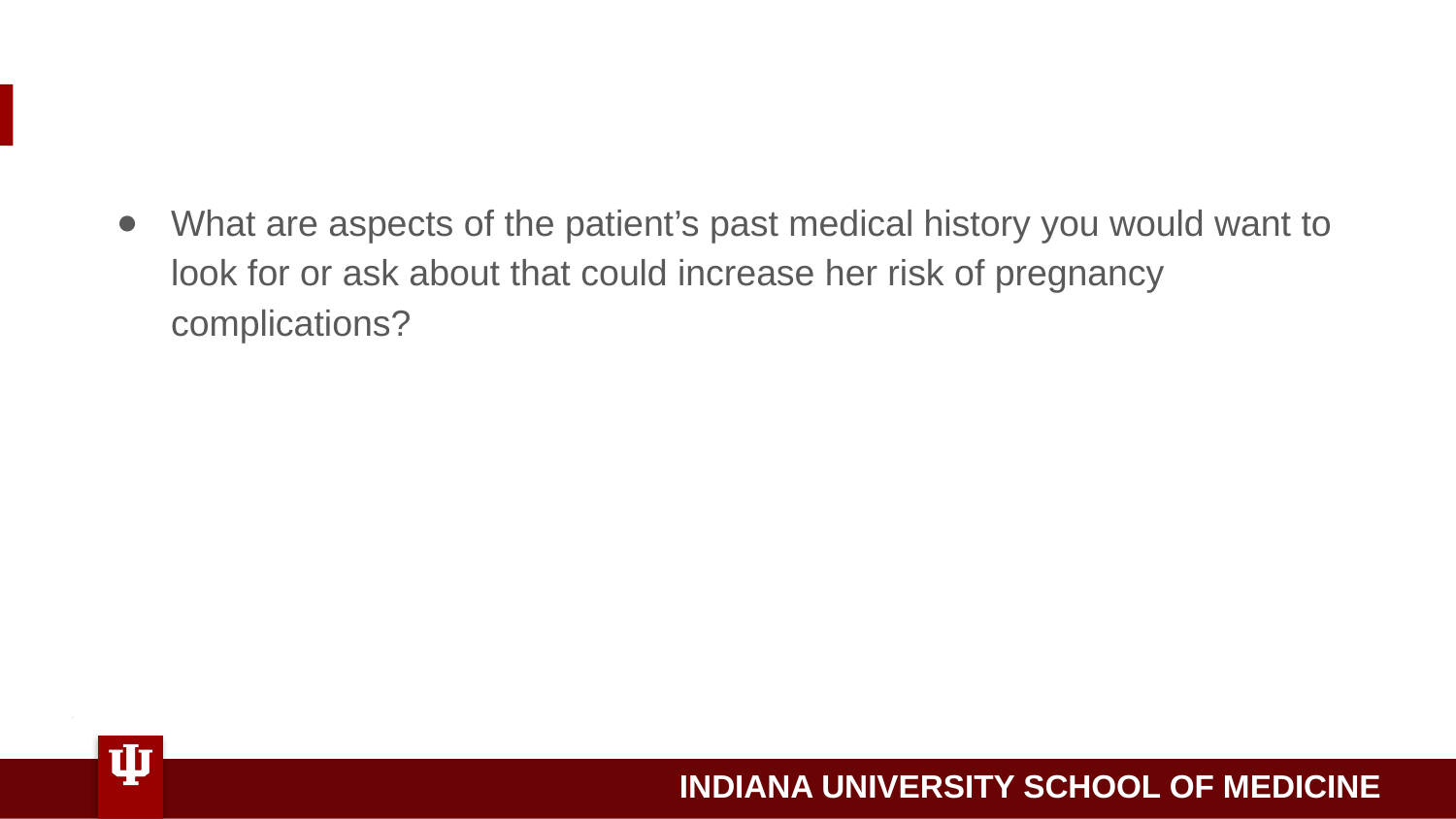

What are aspects of the patient’s past medical history you would want to look for or ask about that could increase her risk of pregnancy complications?

## Slide 14
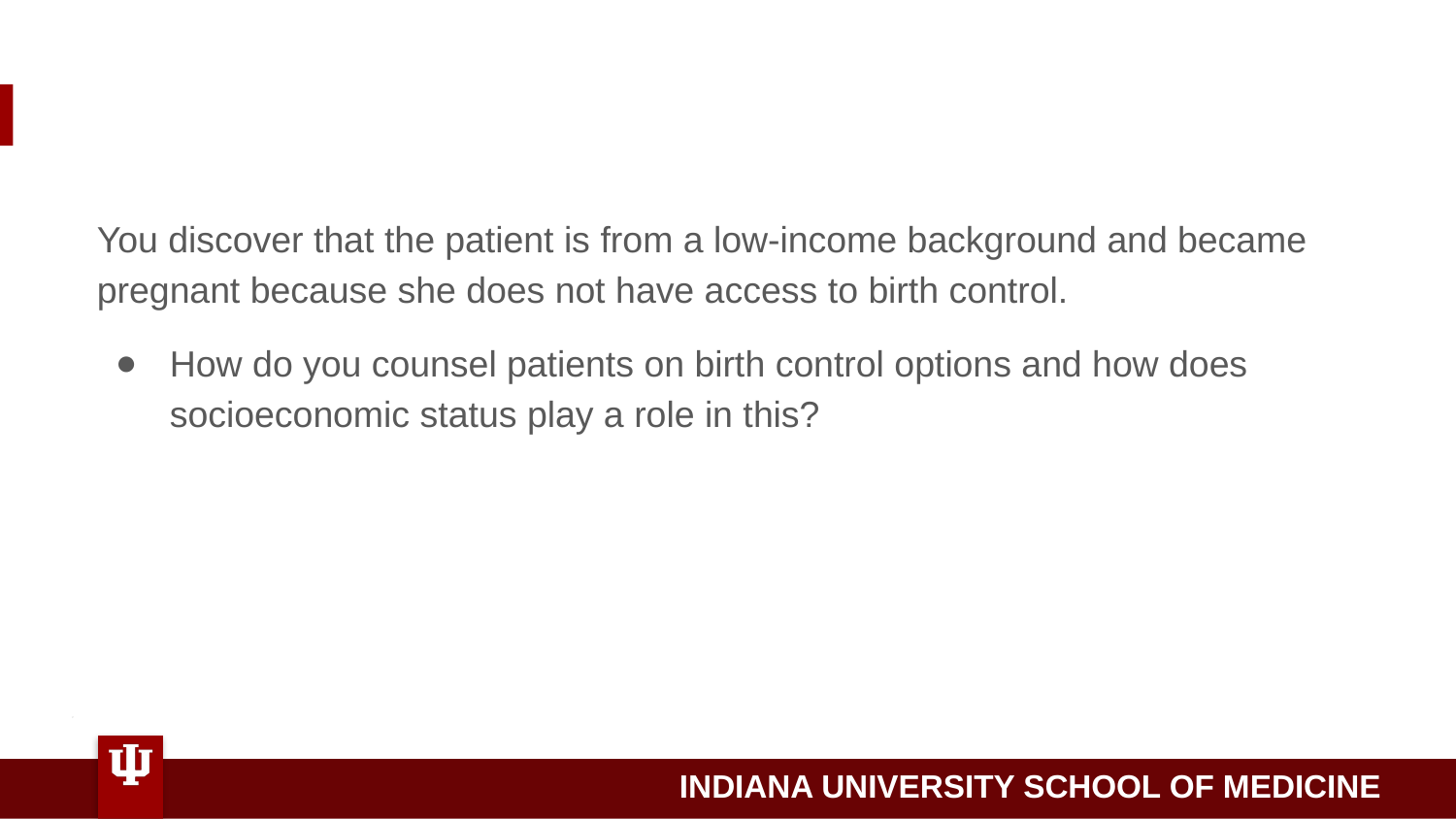

You discover that the patient is from a low-income background and became pregnant because she does not have access to birth control.
How do you counsel patients on birth control options and how does socioeconomic status play a role in this?

## Slide 15
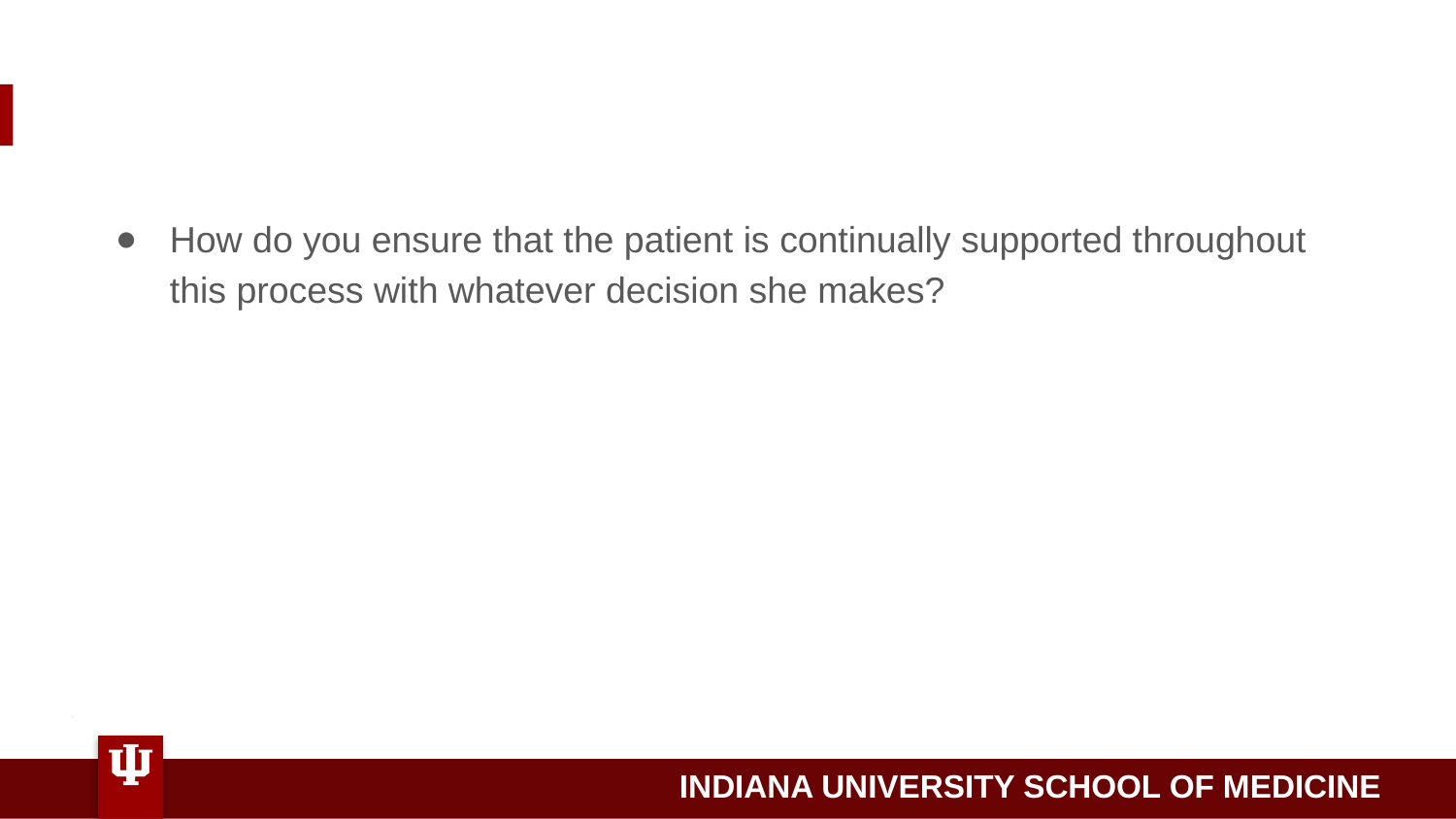

How do you ensure that the patient is continually supported throughout this process with whatever decision she makes?

## Slide 16
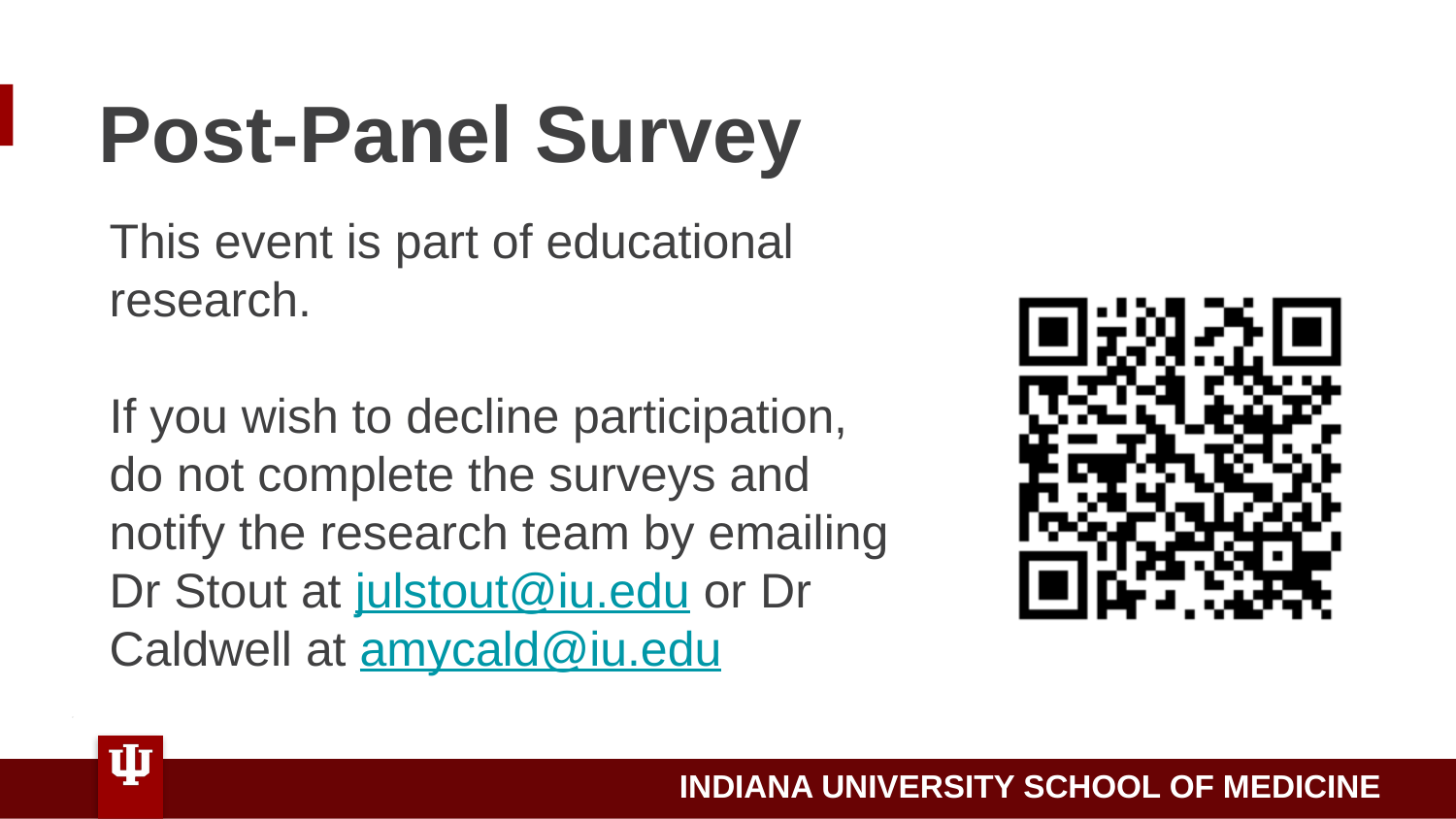

# Post-Panel Survey
This event is part of educational research.
If you wish to decline participation, do not complete the surveys and notify the research team by emailing Dr Stout at julstout@iu.edu or Dr Caldwell at amycald@iu.edu
